# IFI16 restricts SARS-CoV-2 replication by disrupting nucleocapsid-driven phase separation

**DOI:** 10.1038/s42003-026-10363-0

**Published:** 2026-06-01

**Authors:** Ilaria Cislaghi, Sarah Turati, Dalila Vicario, Gloria Griffante, Shikha Chandel, Irene Lo Cigno, Ranieri Bizzarri, Barbara Storti, Tina Ukmar Godec, Milan Zachrdla, Markus Zweckstetter, Lucia Signorini, Kevin Kamau Maina, Serena Delbue, Cinzia Borgogna, Marisa Gariglio

**Affiliations:** 1 Virology Unit, Department of Translational Medicine, University of Piemonte Orientale, Novara, Italy; 2 Department of Surgical, Medical, Molecular Pathology and Critical Care Medicine, University of Pisa, Pisa, Italy; 3 Center for Instrument Sharing of the University of Pisa (CISUP), University of Pisa, Pisa, Italy; 4 Istituto Nanoscienze, CNR, NEST-SNS, Pisa, Italy; 5 German Center for Neurodegenerative Diseases (DZNE), Göttingen, Germany; 6 Department for NMR-based Structural Biology, Max Planck Institute for Multidisciplinary Sciences, Göttingen, Germany; 7 Laboratory of Molecular Virology, Department of Biomedical, Surgical and Dental Sciences, University of Milano, Milano, Italy; 8 Present Address: Department of Public Health and Pediatric Sciences, University of Turin, Turin, Italy

**Keywords:** SARS-CoV-2, Viral host response

## Abstract

IFI16 is an interferon-inducible protein that senses viral DNA and can also restrict RNA virus replication. Here, using IFI16 knockout cellular models, we identify IFI16 as a host restriction factor that limits SARS-CoV-2 replication. Upon SARS-CoV-2 infection, IFI16 relocalizes from the nucleus to the cytoplasm, where it binds both the nucleocapsid protein and the viral genome. This impairs SARS-CoV-2 replication by inhibiting viral RNA–induced condensate formation of the nucleocapsid protein. Finally, we extend our analysis to other human coronaviruses and observe that IFI16 depletion also enhances OC43 replication, whereas it is associated with reduced NL63 replication, highlighting a differential, virus-specific effect of IFI16 on coronavirus infections. Overall, these findings provide mechanistic insights into how the absence of IFI16 creates a cellular environment that supports SARS-CoV-2 replication.

## Abstract (web summary)

IFI16 limits SARS-CoV-2 replication by binding the nucleocapsid protein and viral RNA in the cytoplasm, thereby disrupting condensate formation needed for efficient virus assembly.

## Introduction

Coronaviruses (CoVs) belong to the family *Coronaviridae*, comprising a group of enveloped, positive-sense, single-stranded RNA viruses associated with respiratory and gastrointestinal infections in humans and animals^1,2^. The seven known human coronaviruses (HCoVs) include HCoV-229E, HCoV-OC43, HCoV-NL63, and HCoV-HKU1, which typically cause mild upper respiratory infections, as well as SARS-CoV, MERS-CoV, and SARS-CoV-2, which lead to severe acute respiratory syndromes^1^.

CoV virions are composed of spike (S), envelope (E), and membrane (M) proteins embedded in their viral membrane, with genomic RNA complexed with the nucleocapsid (N) protein forming a helical capsid^3^. The N protein, an RNA-binding protein (RBP) produced at high levels within infected cells, plays multifunctional roles in CoV life cycle^4^. For instance, it facilitates RNA synthesis and translation at early stages of infection and helps assemble genomic RNA into viral RNA-protein complexes during new virion formation^4–6^. To accomplish these functions, beta-CoV N proteins feature a modular architecture with two conserved, folded domains flanked by three intrinsically disordered regions (IDRs)^6^. Interestingly, several IDR-containing RBPs can undergo liquid-liquid phase separation (LLPS), a process that promotes the formation of membraneless organelles (MLOs) to locally concentrate proteins and nucleic acids^7–9^, serving as a regulator of many biological processes, including innate immune reactions^10^. LLPS has been implicated in various stages of the life cycle of several viruses, including SARS-CoV-2 where the N protein operates through LLPS^5,11–14^.

The interferon gamma-inducible protein 16 (IFI16) belongs to the PYHIN protein family and was first reported as a viral DNA sensor mediating innate signaling through TBK-1-dependent interferon (IFN)-β production via STING^15^. It was later found to sense and restrict a range of DNA viruses^16–24^. More recently, IFI16 has also been shown to sense and bind to RNA viral genomes. For instance, IFI16 regulates type I IFN production, thereby inhibiting the replication of Sendai virus (SeV)^24^, porcine reproductive and respiratory syndrome virus (PRRSV)^25^, and influenza A virus (IAV)^26^. Specifically, IFI16 interacts with both negative-sense viral RNA and RIG-I to potentiate RIG-I-mediated IFN-I production, thus inhibiting IAV replication^26^. Moreover, IFI16 binds directly to the genomic RNA of chikungunya virus (CHKV), limiting its replication and maturation^27^. Notably, the IFI16 protein displays an IDR that regulates LLPS following interaction with double-stranded DNA, including that of herpes simplex virus-1 (HSV-1)^28^.

Albeit predominantly nuclear, IFI16 can shuttle between the nucleus and the cytoplasm in response to different stimuli, including infections by DNA and RNA viruses^16,21,26,27,29–31^. Since the majority of RNA viruses, including CoVs, replicate entirely in the cytoplasm^32^, it is possible that cytoplasmic IFI16 may also affect LLPS by binding to RNA viral genomes during replication.

Although the key role of IFI16 in controlling RNA virus replication is well-supported by evidence, its influence on CoV replication, whether directly by modulating viral replication or indirectly by affecting the host innate antiviral response, remains unclear.

In this study, we leveraged IFI16-deficient cellular models to assess how IFI16 influences coronavirus replication. Using SARS-CoV-2 as a model, we show that IFI16 limits infection, as its absence renders cells more permissive to viral replication. We further explore how IFI16 may act in the cytoplasm during infection through interactions with key viral components and by modulating N-associated condensates. Finally, we extend these observations to additional human coronaviruses, highlighting virus-dependent outcomes of IFI16 depletion.

## Results

### Loss of IFI16 promotes SARS-CoV-2 replication

**Fig. 1:**
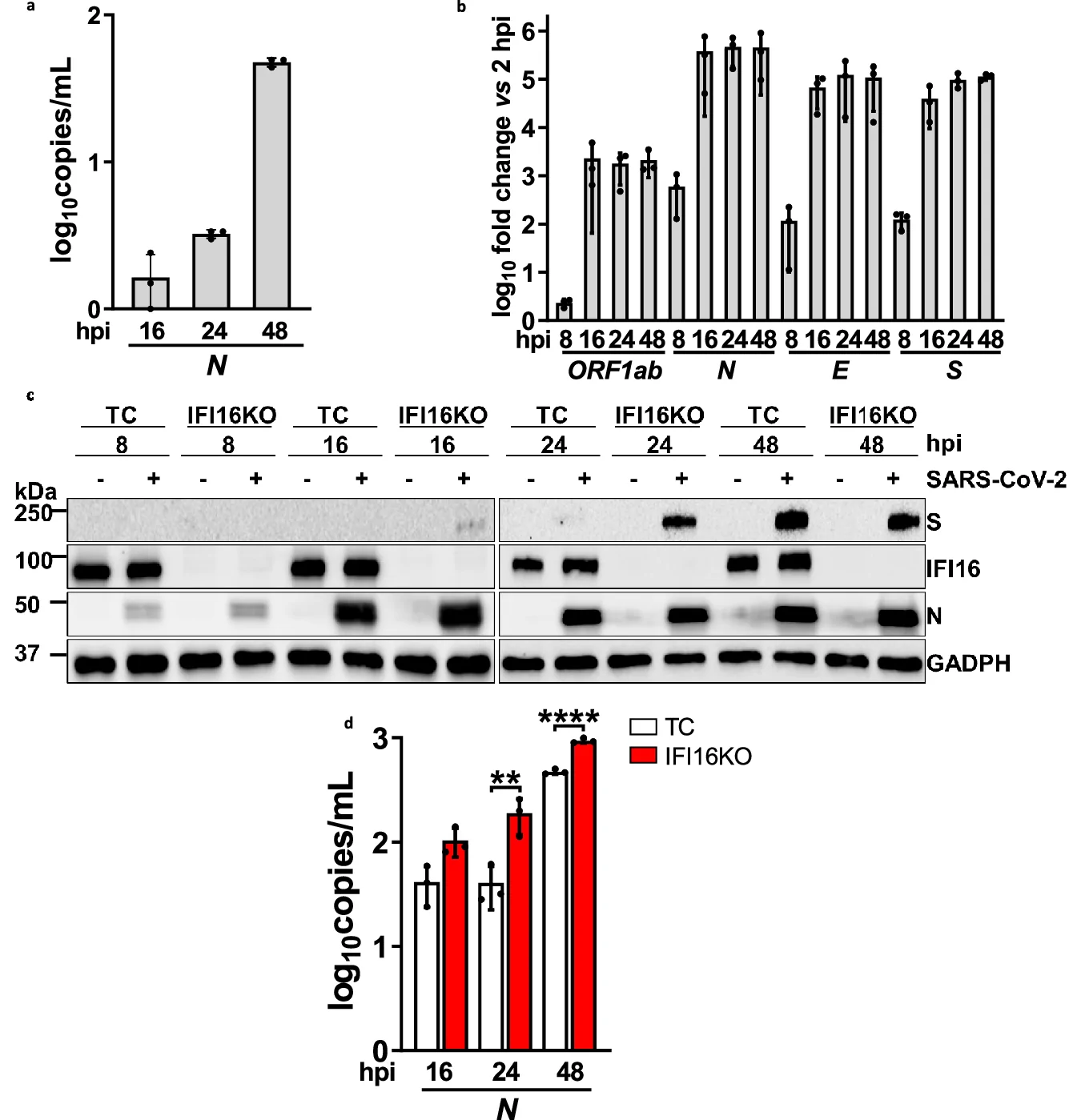
LLC-MK2 cells support SARS-CoV-2 replication and the depletion of IFI16 enhanced its replication rates. **a** LLC-MK2 cells were infected with SARS-CoV-2 at an MOI of 0.5. Cell-free supernatants were harvested at the indicated hpi, and the absolute quantification of viral RNA (N) was determined by ddPCR. Data are presented as mean ± SD (two-way ANOVA, *n* = 3 biological replicates). **b** LLC-MK2 cells were infected with SARS-CoV-2 at an MOI of 0.5. Cell monolayers were harvested at the indicated hpi, and total RNA was extracted. Transcripts of the indicated viral genes were assessed by qRT-PCR. The data are normalized against GAPDH levels and presented as a fold-change relative to 2 hpi. Data are presented as mean ± SD (two-way ANOVA, *n* = 3 biological replicates). **c** TC and IFI16KO cells were mock-infected (−) or infected (+) with SARS-CoV-2 at an MOI of 0.5. At the indicated hpi, total cell extracts were subjected to immunoblot analysis for the detection of the indicated proteins (*n* = 3 biological replicates). **d** TC and IFI16KO cells were infected with SARS-CoV-2 at an MOI of 0.5. Cell-free supernatants were harvested at the indicated hpi, and the absolute quantification of viral RNA was determined by ddPCR. Data are presented as mean ± SD (two-way ANOVA, **p < 0.01, ****p < 0.0001, *n* = 3 biological replicates).

To investigate the role of IFI16 during coronavirus replication, LLC-MK2 cells, a rhesus macaque kidney epithelial cell line selected for its susceptibility to SARS-CoV-2 and other HCoVs, were infected, and viral replication was monitored at early, intermediate, and late stages. Viral replication was first assessed by measuring viral genome copy numbers in the culture supernatants of SARS-CoV-2-infected LLC-MK2 cells using droplet digital PCR (ddPCR), which revealed a progressive increase in extracellular viral RNA (genomic *N*) levels up to 48 h post-infection (hpi) (Fig. 1a). In addition, intracellular viral replication was evaluated by quantifying mRNA levels of the genomic *ORF1ab* and subgenomic *N*, *E*, and *S* genes by RT-qPCR at the same time points. As expected, in SARS-CoV-2-infected cells, intracellular viral RNA levels increased as early as 16 hpi and remained stable up to 48 hpi (Fig. 1b).

We next generated LLC-MK2 clones with stable knockout of the *IFI16* gene, referred to as LLC-MK2 IFI16KO cells (IFI16KO), as well as control cells transduced with non-targeting sgRNAs, named LLC-MK2-transduced control (TC cells) (Supplementary Fig. 1). Protein expression levels of both N and S proteins in IFI16KO cells infected (+) or not (−) with SARS-CoV-2 were assessed by Western blotting (Fig. 1c). The N protein was detectable as early as 8 hpi, with its expression markedly increasing at 16 hpi and continuing to rise at later time points in both TC and IFI16KO cells (Fig. 1c). The S protein was already detectable at 16 hpi and to a higher extent at 24 and 48 hpi in SARS-CoV-2-infected IFI16KO cells, while in TC cells similarly infected it could only be detected at 48 hpi. Intriguingly, IFI16 protein expression levels in TC cells remained unchanged upon infection with SARS-CoV-2 (Fig. 1c).

Next, the replication rate of SARS-CoV-2 was assessed in infected IFI16KO *vs* TC culture supernatants by ddPCR at different time points post-infection (pi). As shown in Fig. 1d, the copy number of SARS-CoV-2 viral genome was significantly higher in IFI16KO cells compared to TC cells at both 24 and 48 hpi (*p* = 0.0024 and *p* < 0.0001, respectively).

**Fig. 2:**
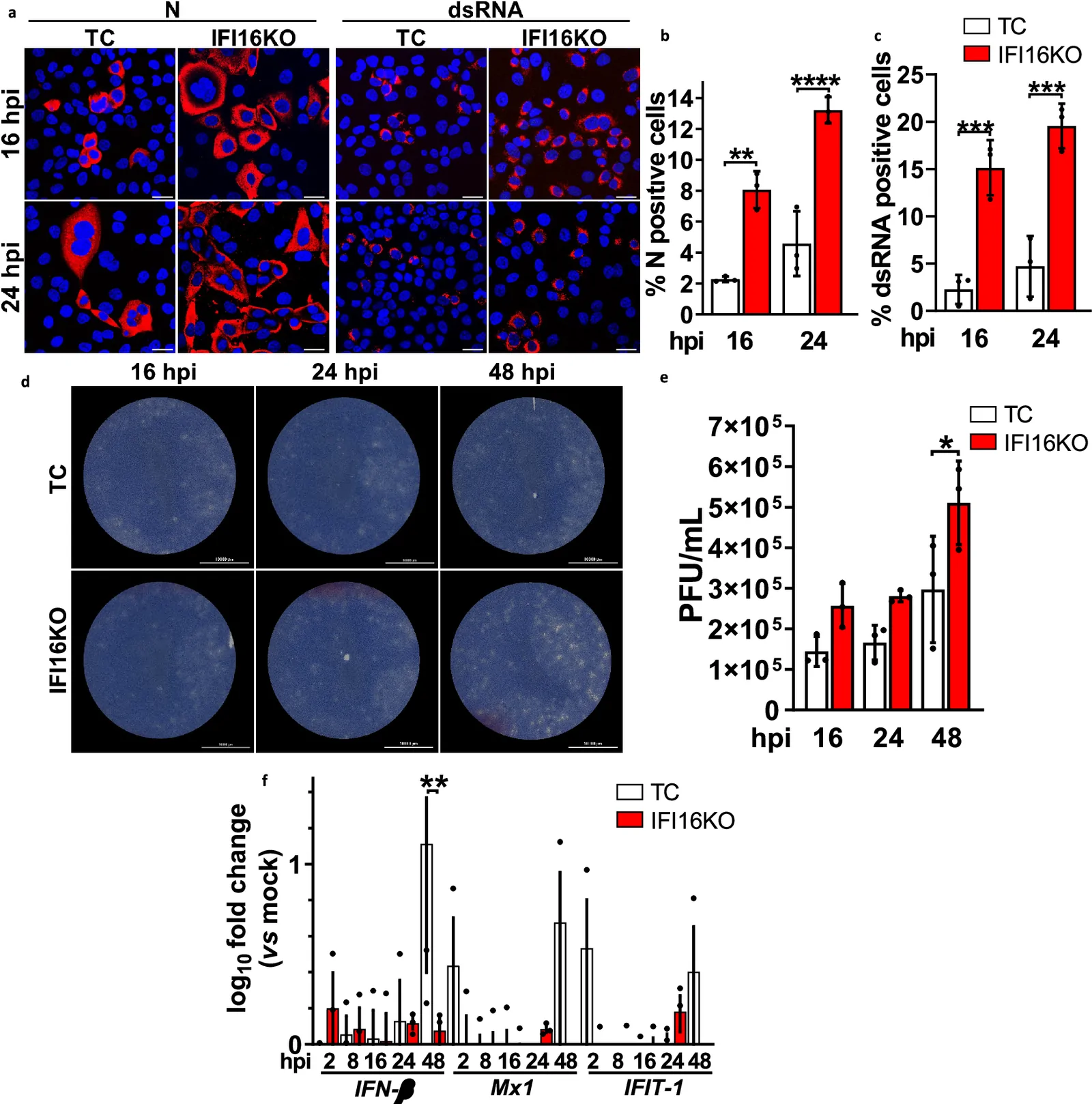
IFI16 restricts SARS-CoV-2 replication in LLC-MK2 cells with minimal IFN-β/ISG induction. **a** TC and IFI16KO cells were infected with SARS-CoV-2 at an MOI of 0.5. At the indicated hpi cells were processed for immunofluorescence analysis for N (left panel) or dsRNA (right panel), both labeled in red. Nuclei were visualized by DAPI (blue). Images were captured by confocal microscopy and are representative of six different fields from three independent experiments. Scale bars: 25 μm. **b**, **c** Images shown in (**a**) were quantified using THUNDER Imager 3D Live Cell (Leica Microsystems). Data are presented as mean ± SD of the percentage of cells positive for N or dsRNA over total cells. The analysis was performed on six different fields from three independent experiments (two-way ANOVA test). **p < 0.01, ***p < 0.001, ****p < 0.0001). All original images used for quantification are available from the corresponding author upon request. **d** TC and IFI16KO cells were infected with SARS-CoV-2 (MOI 0.5). Supernatants were collected at 16, 24, and 48 hpi and titrated by standard plaque assay analysis on VeroE6 cells. The cytopathic effect (CPE) in cultures was monitored by optical microscopy to demonstrate the extent of infection. Representative images of three independent experiments are shown. **e** Plaques were counted at 48 hpi using a microplate reader Cytation 5 (BioTek) and expressed as plaque forming unit (PFU)/mL. Data are presented as mean ± SD (two-way ANOVA test. *p < 0.05, *n* = 3 biological replicates). Scale bar: 10.000 μm. **f** TC and IFI16KO cells were infected with SARS-CoV-2 at an MOI of 0.5. Cell monolayers were harvested at the indicated hpi, and total RNA was extracted. Transcripts of the indicated viral genes were assessed by qRT-PCR. The data were normalized against GAPDH levels and presented as fold change relative to mock-infected cells. Data are presented as means ± SD (two-way ANOVA; **p < 0.01, *n* = 3 biological replicates).

We also assessed N protein and double-stranded RNA (dsRNA) expression levels, an intermediate of viral replication, by immunofluorescence analysis in both TC and IFI16KO cells infected with SARS-CoV-2 (Fig. 2a). High resolution confocal microscopy analysis demonstrated that, as expected, in IFI16KO-infected cells the percentage of N-positive cells was significantly higher than that observed in TC cells at both 16 and 24 hpi (8.1% vs 2.3%; *p* = 0.0011; 13.2% vs 4.6%; *p* < 0.0001, respectively) (Fig. 2b).

As a readout of viral infection, we monitored dsRNA accumulation using J2 antibody staining by immunofluorescence. In SARS-CoV-2-infected IFI16KO cells, the number of dsRNA-positive cells was significantly higher compared to TC cells at both 16 and 24 hpi (15.1% vs 2.3%; *p* = 0.0006; 19.5% vs 4.7%; *p* = 0.0002) (Fig. 2c).

The higher replication rate of SARS-CoV-2 in IFI16-depleted *vs* TC cells was confirmed by plaque assay titration of SARS-CoV-2 infectious viral particles in Vero E6 cells using cell culture supernatants from TC or IFI16KO infected cells. As shown in Fig. 2d, e, the number of plaques obtained with the supernatants harvested at 48 hpi and derived from IFI16KO-infected cells significantly exceeded those from TC-infected cells (511,000 *vs* 297,000 PFU/mL; *p* = 0.0139). In line with these results, ddPCR quantification of viral RNA also supported increased SARS-CoV-2 replication upon IFI16 loss, showing higher vRNA levels as early as 24 hpi and a more pronounced difference at 48 hpi (Fig. 1d). Collectively, these findings support an antiviral role for IFI16 during SARS-CoV-2 infection.

We next asked whether loss of IFI16 altered the innate immune response to SARS-CoV-2, which could indirectly affect viral replication. We therefore quantified IFN-β mRNA levels by RT–qPCR in TC and IFI16KO cells following infection (Fig. 2f), together with the IFN-stimulated genes MX1 and IFIT1. In our experimental model, infection did not significantly induce IFN-β or these downstream genes. Notably, the modest late increase in IFN-β transcripts observed in infected TC cells was significantly reduced in IFI16KO cells (*p* = 0.0063).

**Fig. 3:**
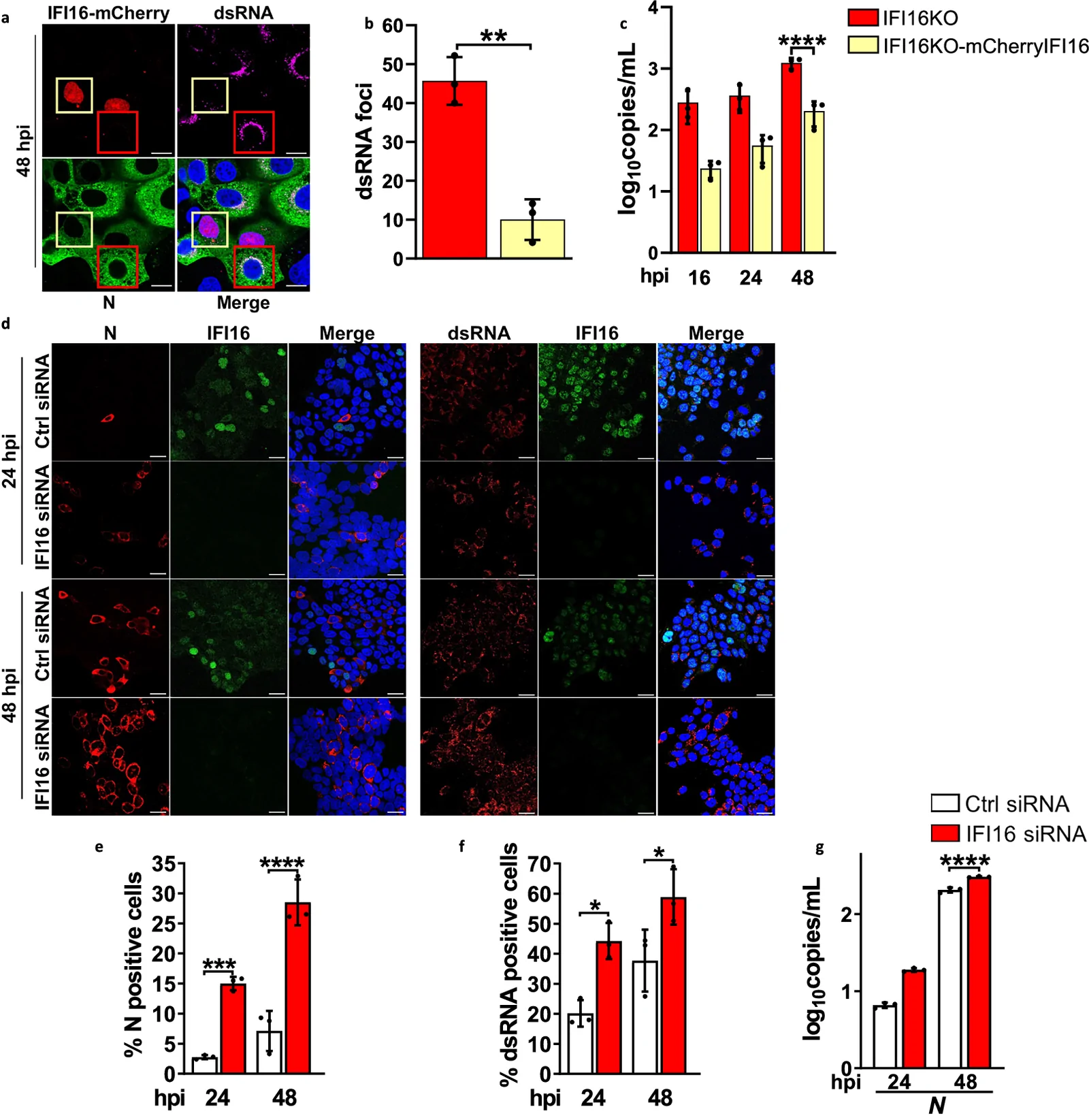
IFI16 complementation reverses the IFI16KO phenotype, and IFI16 depletion increases SARS-CoV-2 replication in Calu-3 cells. **a** IFI16KO cells were transfected with the mCherryIFI16 plasmid for 24 h and then infected with SARS-CoV-2 (MOI 0.5) for another 48 h. At the indicated hpi cells were processed for immunofluorescence analysis for dsRNA (pink) and N (green). Nuclei were visualized by DAPI (blue). Images were captured by confocal microscopy and are representative of six different fields from three independent experiments. Scale bars: 10 μm. **b** Images shown in (**a**) were quantified using ImageJ software. Data are presented as mean ± SD of the number of dsRNA foci in transfected versus non-transfected cells from three independent experiments (unpaired Student’s *t*-test, **p < 0.01). **c** IFI16KO cells were transfected with the mCherryIFI16 plasmid or left untransfected for 24 h and then infected with SARS-CoV-2 (MOI 0.5) for another 8, 16, 24, and 48 h. Cell-free supernatants were harvested at the indicated hpi, and the absolute quantification of viral RNA was determined by ddPCR. Data are presented as mean ± SD (two-way ANOVA, ****p < 0.0001, *n* = 3 biological replicates). **d** Calu-3 control siRNA and IFI16 siRNA cells were infected with SARS-CoV-2 at an MOI of 0.5. At the indicated hpi, cells were processed for immunofluorescence analysis for N or dsRNA, both labeled in red. Nuclei were visualized by DAPI (blue). Images were captured by confocal microscopy and are representative of six different fields from three independent experiments. Scale bars: 25 μm. **e**, **f** Images shown in (**d**) were quantified using THUNDER Imager 3D Live Cell (Leica Microsystems). Data are presented as mean ± SD of the percentage of cells positive for N or dsRNA over total cells. The analysis was performed on six different fields from three independent experiments (two-way ANOVA test. *p < 0.05, ***p < 0.001, ****p < 0.0001). All original images used for quantification are available from the corresponding author upon request. **g** Calu-3 control siRNA and IFI16 siRNA cells were infected with SARS-CoV-2 at an MOI of 0.5. Cell-free supernatants were harvested at the indicated hpi, and the absolute quantification of viral RNA was determined by ddPCR. Data are presented as mean ± SD (two-way ANOVA, ****p < 0.0001, *n* = 3 biological replicates).

To determine whether the observed phenotype was specifically attributable to IFI16 deficiency, we performed complementation experiments by transfecting IFI16KO cells with a plasmid encoding IFI16 fused to mCherry prior to SARS-CoV-2 infection. As shown in Fig. 3a, immunofluorescence analysis at 48 hpi revealed a marked reduction in dsRNA foci—indicative of viral replication intermediates—in IFI16-rescued cells (IFI16KO–mCherryIFI16) compared with IFI16KO cells (*p* = 0.0016; Fig. 3b). This reduction was accompanied by a significant decrease in viral RNA levels at 48 hpi, as quantified by ddPCR (*p* < 0.0001; Fig. 3c), indicating that reconstitution of IFI16 restores its antiviral activity against SARS-CoV-2.

To extend these findings to a widely used model of human respiratory epithelium, we silenced IFI16 in Calu-3 lung epithelial cells prior to SARS-CoV-2 infection. Cells transfected with control or IFI16-targeting siRNA (ctrl siRNA and IFI16 siRNA, respectively) were analyzed at 24 and 48 hpi. Immunofluorescence staining for the viral N protein and dsRNA upon IFI16 silencing compared with control cells showed a significant increase in both N- [15% vs 2.8%; *p* = 0.0008; 28.5% vs 7,1%; *p* < 0.0001 (Fig. 3d, e)] and dsRNA-positive cells [44.2% *vs* 20.1%; *p* = 0.0112; 58.9% *vs* 37.7%; *p* = 0.0215) (Fig. 3d, f)] consistent with enhanced viral replication. Consistently, quantification of viral genome copies in culture supernatants by ddPCR revealed significantly higher viral RNA levels in IFI16 siRNA–treated cells compared to ctrl siRNA cells at 48 hpi (*p* < 0.0001, Fig. 3g). Collectively, these results demonstrate that IFI16 restricts SARS-CoV-2 replication and that its loss enhances viral propagation. Importantly, in the LLC-MK2 cellular model, we did not observe a concomitant induction of IFN-β or representative ISGs associated with the antiviral phenotype, indicating that under these experimental conditions, the restrictive effect of IFI16 is unlikely to be primarily driven by type I IFN induction.

### IFI16 relocalizes to the cytoplasm upon SARS-CoV-2 infection and associates with N protein and viral genomic RNA

**Fig. 4:**
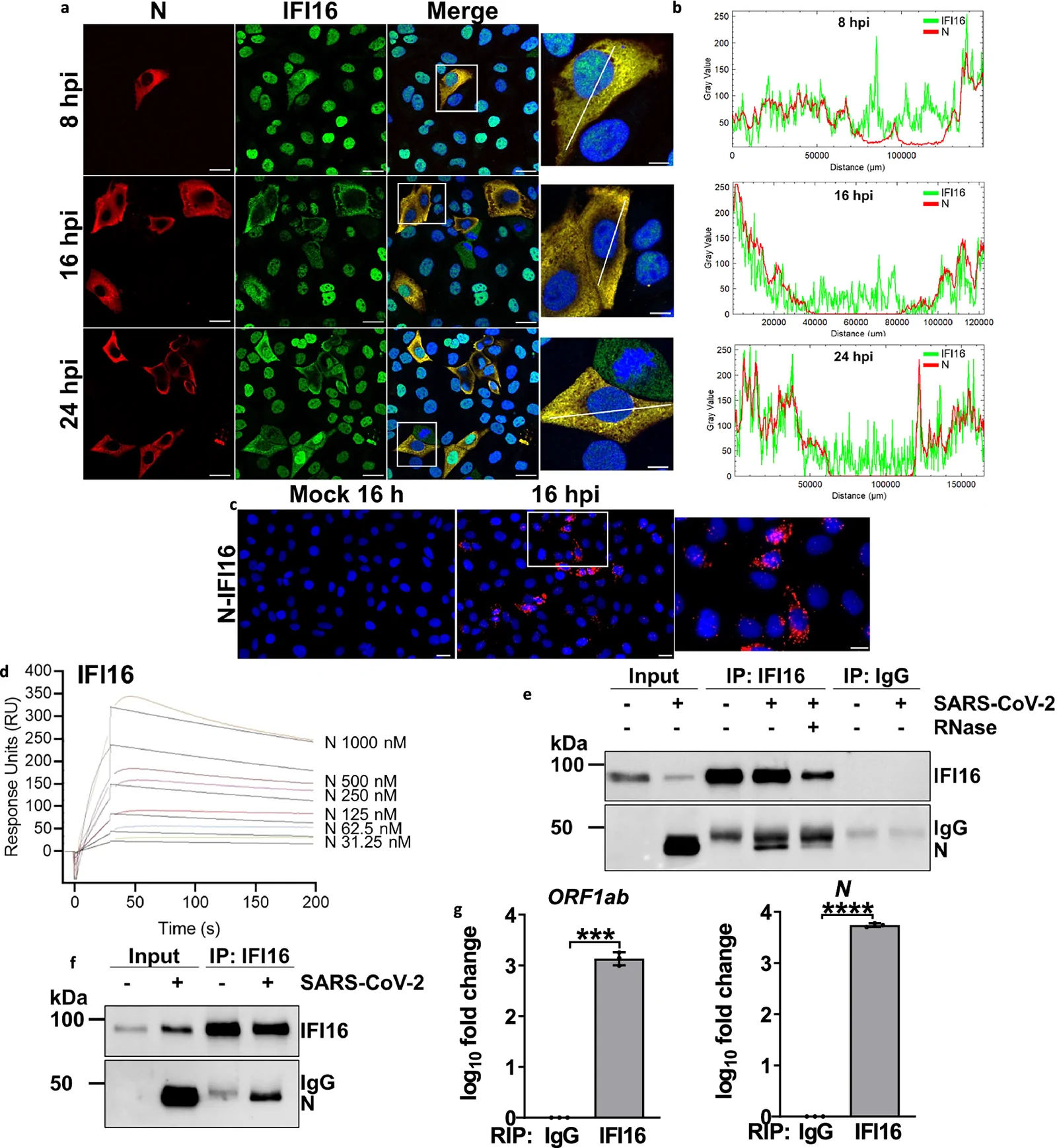
The IFI16 protein translocates to the cytoplasm upon HCoV infection and binds to the N protein and the viral genome. **a** TC cells were infected with SARS-CoV-2 (MOI 0.5), and at the indicated hpi cells were processed for immunofluorescence analysis for IFI16 (green) and N (red). Nuclei were visualized by DAPI (blue). Images were captured by confocal microscopy and are representative of six different fields from three independent experiments. Scale bars: 25 μm. The images in the last column correspond to the magnified areas highlighted by a white square in the merged image. Scale bars: 10 μm. **b** The fluorescence intensity profiles of IFI16 (green) and N (red) across different areas of the magnified cells from (**a**) (far-right column), as indicated by the white line, are plotted. The graphs show the distribution and colocalization of N and IFI16 proteins as determined by ImageJ/FiJi. **c** TC cells were infected with SARS-CoV-2 (MOI 0.5), and at 16 hpi, cells were processed for PLA analysis with anti-IFI16 or anti-N antibodies. Nuclei were visualized by DAPI (blue). Images were acquired using a Zeiss AxioScan.Z1 and are representative of three independent experiments. Scale bars: 20 μm. The image shown in the lower panel corresponds to the magnified area highlighted by a white square in the image above. Scale bar: 10 μm. **d** Representative SPR analysis of IFI16 binding to the N protein from SARS-CoV-2. Recombinant IFI16 was immobilized on a CM5 sensor chip surface, and increasing concentrations of recombinant N protein (from 31.25 to 1000 nM) diluted in running buffer were injected over immobilized IFI16. IFI16 binding to the N protein of SARS-CoV-2 displays an equilibrium dissociation constant (*K*_D_) of 2.284 × 10^−8^M. **e** TC cells were mock-infected (−) or infected (+) with SARS-CoV-2 (MOI 3). Cells were harvested at 16 hpi and cytoplasmic cell lysates were subjected to immunoprecipitation using anti-IFI16 or anti-IgG antibodies. Immunoprecipitates, with or without RNase treatment, were analyzed by immunoblotting. The additional band observed in the IP lanes probed with anti-N represents the IgG heavy chain from the anti-IFI16 immunoprecipitating antibody, detected by the species-specific secondary antibody used in the Western blot. One representative Western blot from three independent experiments is shown. **f** TC cells were mock-infected (−) or infected (+) with SARS-CoV-2 (MOI 3). At 16 hpi, cells were subjected to UV irradiation at 250 mJ cm^-2^ for crosslinking and then harvested. Cytoplasmic cell lysates were subjected to immunoprecipitation using an anti-IFI16 antibody. Immunoprecipitates were analyzed by immunoblotting. One representative Western blot from three independent experiments is shown. **g** SARS-CoV-2 RNA from the immunoprecipitates obtained as described in panel (**f**) was extracted, and genomic viral transcripts of the indicated viral genes were analyzed by qRT-PCR. The data were presented as log_10_ fold induction relative to IgG control (set to 0). Data are presented as mean ± SD (Paired *t* test, ***p < 0.001, ****p < 0.0001, *n* = 3 biological replicates).

Despite typically residing in the nucleus, IFI16 can translocate to the cytoplasm upon infection with a variety of DNA viruses, including herpes simplex virus 1 (HSV-1)^19,33,34^, human cytomegalovirus (hCMV)^16,21^, Kaposi’s sarcoma-associated herpesvirus (KSHV)^35^, and Epstein-Barr virus (EBV)^36^. To determine whether IFI16 also re-localizes to the cytoplasm following HCoV infection, we performed co-staining of SARS-CoV-2-infected-TC cells for both the IFI16 and N proteins, using the latter as a marker of viral infection. Upon SARS-CoV-2 infection, IFI16 translocated from the nucleus to the cytoplasm of N-positive cells, peaking at 16 hpi (Fig. 4a). Quantitative analysis of the intensity and cellular distribution of N and IFI16 fluorescent signals confirmed cytoplasmic colocalization of the two proteins at all time points (Fig. 4b), suggesting a role for IFI16 in the antiviral response during the early stages of the viral replication cycle. Consistently, in SARS-CoV-2-infected Calu-3 cells, we observed IFI16 translocation to the cytoplasm together with clear colocalization between IFI16 and N (Supplementary Fig. 2), in line with the findings obtained in the LLC-MK2 model.

The close proximity between N and IFI16 in TC-infected cells was confirmed by proximity ligation assay (PLA) using specific antibodies. PLA fluorescent puncta were observed in the cytoplasm of infected cells at 16 hpi (22.2 PLA puncta/cell ± 0.19), but not in mock-infected cells, thus confirming the specificity of the staining (Fig. 4c). The specificity of the binding between IFI16 and the N protein of SARS-CoV-2 was further evaluated by surface plasmon resonance (SPR) analysis. To this end, recombinant IFI16 was immobilized on a CM5 sensor chip and then probed with increasing concentrations of the recombinant N protein of SARS-CoV-2—ranging from 31.25 to 1000 nM. The resulting SPR sensorgrams revealed significant binding between IFI16 and the N protein, with an equilibrium dissociation constant (K_D_) of 2.284 × 10^−8^M (Fig. 4d). In addition, we performed immunoprecipitation assays with anti-IFI16 polyclonal antibodies using cytosolic extracts of SARS-CoV-2-infected TC cells collected at 16 hpi. Anti-IFI16 antibodies, but not anti-IgG antibodies, co-immunoprecipitated the N protein in infected cells (Fig. 4e), confirming their interaction in our cellular model. The IFI16–N complex persisted after RNase treatment, indicating that the interaction does not require viral RNA. Notably, RNase treatment reduced the amount of co-immunoprecipitated N protein, which is likely attributable to altered recovery of IFI16-containing complexes following disruption of RNA-dependent IFI16 assemblies (Fig. 4e).

Since IFI16 restricts viral replication by binding to the genomic RNA of influenza A virus (IAV)^26^ and Chikungunya virus (CHIKV)^27^, we thought possible that, upon translocating from the nucleus to the cytoplasm, IFI16 might act as an RNA-binding protein (RBP) interacting with the SARS-CoV-2 genome. To test this hypothesis, we performed RNA immunoprecipitation (RIP) analysis using cytosolic lysates from SARS-CoV-2-infected TC cells harvested at 16 hpi, which had been UV crosslinked to “freeze” any intermolecular RNA/protein interactions. The immunoprecipitation was performed using polyclonal antibodies directed against either IgG (data not shown) or IFI16 and validated by Western blot analysis (Fig. 4f). Subsequently, co-precipitated viral RNA was extracted from the immunoprecipitates, and the RNA expression levels of genomic *ORF1ab* and *N* genes were determined by RT-qPCR. As shown in Fig. 4g, there was a significant enrichment of SARS-CoV-2 RNAs in the IFI16-IP compared to the IgG-IP, confirming the interaction between IFI16 and viral RNA. All viral genes analyzed showed increased expression in the infected-cell pull-down, with a statistically significant 3-fold increase for the genomic *ORF1ab* gene (*p* = 0.0006) and a 4-fold upregulation for the genomic *N* gene (*p* < 0.0001). Fold enrichment values are reported on a linear scale, while data are plotted as log10-transformed values relative to the IgG control (set to 0). Thus, upon translocation to the cytoplasm in SARS-CoV-2-infected cells, IFI16 forms a cytoplasmic complex with the N protein and the viral genome.

### IFI16 interferes with N/vRNA-driven phase separation and modulates N condensate architecture during infection

**Fig. 5:**
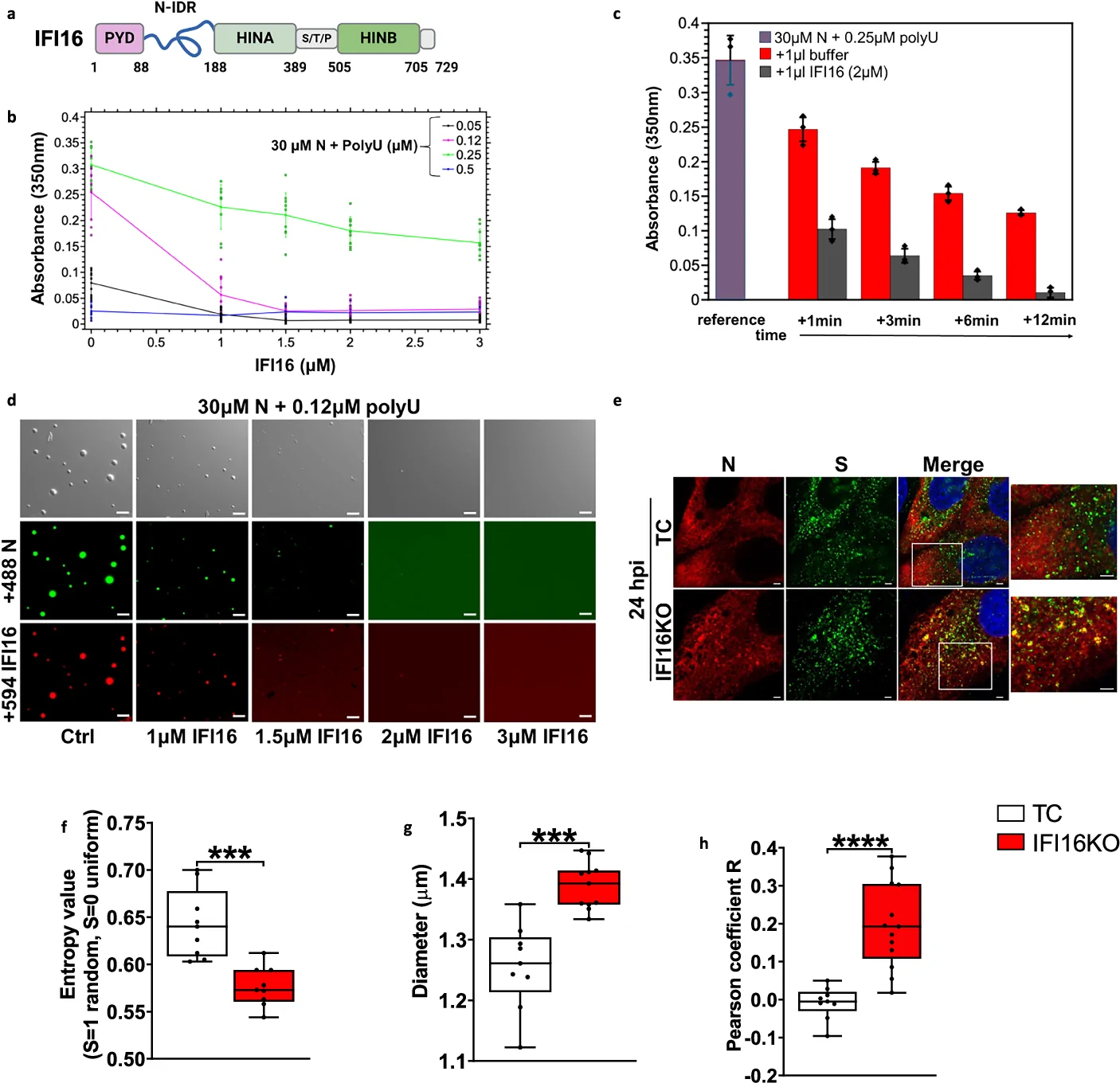
The IFI16 protein disrupts the formation of N/vRNA LLPS condensates both in biochemical assay and infected cells. **a** Schematic representation of the IFI16 protein. The following domains are highlighted: pyrin domain (PYD), hematopoietic interferon-inducible nuclear (HIN) domains A and B, and intrinsically disordered region (IDR). Created with BioRender.com licensed under CC BY 4.0. https://BioRender.com/yi2pf5c. **b** Turbidity measurements of N protein (30 μM) in the presence of increasing concentrations of polyU (0.05–0.5 μM) and IFI16 (1–3 μM), in 20 mM NaP buffer, pH 7.5. Data are presented as mean ± SD (*n* = 3 independent samples and 3 measurements per sample). **c** Turbidity experiments showing the effect of IFI16 on the dissolution of N/RNA condensates monitored over time. The purple bar represents the reference turbidity value of the N/RNA condensate sample. The experiment included the addition of 1 μl of buffer (red bars), as a control, and 1 μl of IFI16 (2 μM, grey bars) to examine its effect on the N/RNA condensates (*n* = 3 independent samples). **d** DIC and fluorescence images demonstrating the inhibitory effect of IFI16 on N/RNA condensates. For fluorescence imaging, samples were mixed with Alexa 488-labeled N protein (green) and Alexa 594-labeled IFI16 protein (red). Scale bars: 10 μm. **e** TC and IFI16KO cells were infected with SARS-CoV-2 (MOI 0.5) and processed for immunofluorescence analysis for N (red) and S (green). Nuclei were visualized by DAPI (blue). Images were captured by Airyscan microscopy and are representative of eight different fields from three independent experiments. Scale bars: 2 μm. The pictures shown in the right-hand panels correspond to the magnified areas highlighted by the white squares in the merged images. Scale bars: 2 μm. **f** N protein aggregation in TC and IFI16KO cells from the images shown in (**e**) was quantified using ImageJ/FiJi. Data are presented as mean ± SD of the entropy value, where *S* = 1 indicates randomness, while *S* = 0 denotes uniformity. The analysis was performed on nine different fields in a representative experiment (unpaired Student’s *t*-test, ***p < 0.001). **g** Diameter of N protein condensates in TC and IFI16KO cells from the images shown in (**e**) was quantified using ImageJ/FiJi. Data are presented as mean ± SD of the diameter of the condensates in μm. The analysis was performed on nine different fields in a representative experiment (unpaired Student’s *t*-test with Welch’s correction, ***p < 0.001). **h** Pearson colocalization coefficients (R) of N (red) and S (green) in TC and IFI16KO cells were quantified using ImageJ/FiJi. R values close to 1 indicate a high degree of colocalization. Data are presented as mean ± SD of R. The analysis was performed on nine different fields in a representative experiment (Mann–Whitney test, ****p < 0.0001). All original images used for quantification are available from the corresponding author upon request.

Recent reports, including one by our co-authors, have shown that the N protein undergoes LLPS in the presence of viral genomic RNA, and that the formation of these RNA-protein condensates enhances the efficiency of viral RNA transcription and virion assembly^11,37,38^. Given the role of the N protein in viral replication and packaging^4^, and considering that host RBPs can influence its function—primarily by modulating the LLPS of N/vRNA condensates, which act as a scaffold for viral replication while facilitating the assembly of viral replication complexes^7,12,38^—we postulated that IFI16, through its IDR (Fig. 5a), might interfere with the LLPS process of the N protein. To test this hypothesis, we performed turbidity measurements with N protein solutions in the presence of increasing concentrations of polyU, used as a substitute for viral RNA, and IFI16 (Fig. 5b and Supplementary Fig. 3). The addition of 1 μM IFI16 markedly reduced the turbidity of the solution, and this reduction continued progressively with increasing concentration of IFI16 (Fig. 5b and Supplementary Fig. 3), indicating that IFI16 exerts a dose-dependent suppressive effect on the formation of N/polyU condensates. To assess the effect of IFI16 on the dissolution of these condensates, we monitored the turbidity of N protein solutions over time following the addition of 0.25 μM polyU and 2 μM IFI16 (Fig. 5c and Supplementary Fig. 4). The addition of IFI16 led to a significant, time-dependent reduction in N/polyU condensate formation. Consistently, the absorbance of the N protein solution decreased significantly within 1 min after adding IFI16 and continued to decline, approaching zero by 12 min (Fig. 5c). This time-dependent dissolution effect of IFI16 on N/polyU condensates was further corroborated by differential interference contrast (DIC) and fluorescence microscopy (Supplementary Fig. 4). Furthermore, DIC and fluorescence images revealed co-recruitment of both N and IFI16 proteins into N/polyU condensates and confirmed the concentration-dependent inhibitory effect of IFI16 (Fig. 5d). Thus, IFI16 plays an inhibitory role in the formation of N/vRNA LLPS condensates.

Based on these findings, we anticipated alterations in N condensate formation in IFI16-depleted cells compared to their normal counterpart during SARS-CoV-2 infection. Super-resolution microscopy of infected IFI16KO cells at 24 hpi revealed that the N protein predominantly localized in perinuclear clusterized structures, while in TC cells at the same time point, it showed a more homogeneous cytoplasmic distribution (Fig. 5e).

To quantify the spatial texture differences of N protein in the cytoplasm of IFI16KO and TC cells, we measured their grayscale histogram entropy^39^. Higher entropy values indicate a more homogeneous distribution, whereas lower values suggest a more organized localization. We found a statistically significant decrease in entropy in IFI16KO *vs* TC cells (S = 0.58 and 0.64, respectively; *p* = 0.0002) (Fig. 5f), consistent with the observed cytoplasmic presence of N condensates. This finding was further confirmed by granulometric analysis of the super-resolution images^40^, which revealed a significantly larger average granulometric diameter of pixel distributions in IFI16KO compared to TC cells (1.39 mm *vs* 1.26 mm, respectively; *p* = 0.0003) (Fig. 5g).

When co-immunostained with different fluorophores, viral N and S proteins showed colocalization in IFI16KO cells compared to TC cells at 24 hpi, especially within N condensates. The extent of colocalization was quantified using Pearson’s correlation coefficient (R), which measures the linear correlation between the fluorescence intensities of two proteins in each pixel of an image. The R value in IFI16KO cells was significantly higher than that seen in TC cells (0.19 *vs* -0.005, respectively; *p* < 0.0001) (Fig. 5h). Thus, IFI16KO cells displayed earlier formation of large perinuclear N-enriched condensates and increased N–S colocalization, consistent with enhanced formation of assembling virions, compared to TC cells. Quantitative image analyses (entropy and granulometry) further supported differences in the size and spatial organization of N-positive structures between IFI16KO and TC cells.

### IFI16 differentially modulates the replication of the low-pathogenic coronaviruses OC43 and NL63

**Fig. 6:**
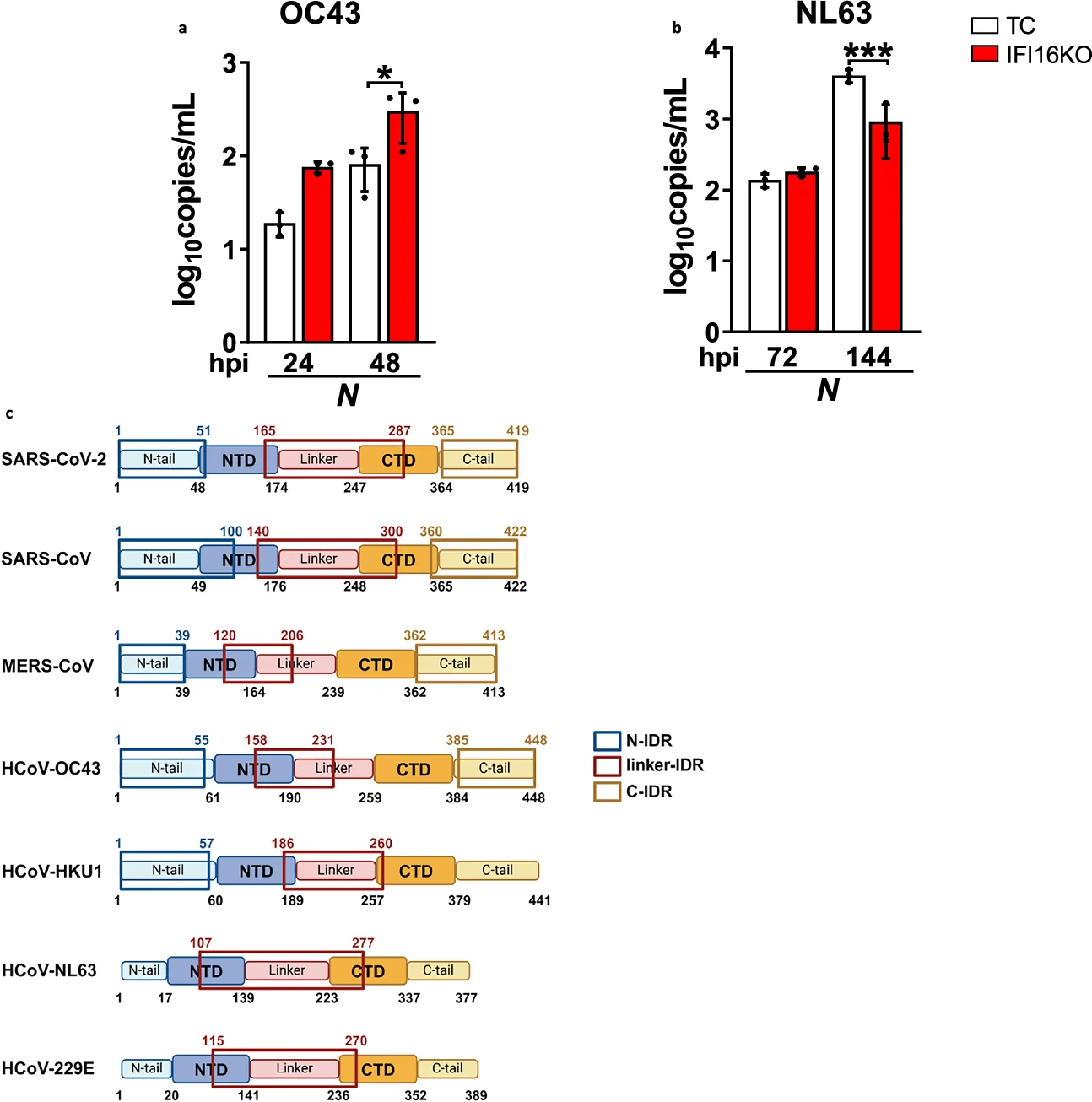
The IFI16 protein also impairs OC43 replication but promotes NL63 replication. TC and IFI16KO cells were infected with OC43 (**a**) or NL63 (**b**) at an MOI of 0.01 and 1, respectively. Cell-free supernatants were harvested at the indicated hpi, and the absolute quantification of viral RNA was determined by ddPCR. Data are presented as mean ± SD (two-way ANOVA, *p < 0.05, ****p < 0.0001, *n* = 3 biological replicates). **c** Schematic representation of the N proteins of the seven HCoVs. The following domains are highlighted: N-terminal domain (NTD), C-terminal domain (CTD), and intrinsically disordered region (IDR). Created with BioRender.com licensed under CC BY 4.0. https://BioRender.com/6hvfilx.

To assess whether IFI16 also modulates the replication of low-pathogenic HCoVs, we infected IFI16KO and TC cells with the betacoronavirus OC43 and the alphacoronavirus NL63, and quantified viral genome copies in culture supernatants by ddPCR. For OC43, viral replication was significantly higher in IFI16KO cells at 48 hpi (*p* = 0.0276) (Fig. 6a), indicating that IFI16-deficient cells favor increased OC43 replication, consistent with our observations for SARS-CoV-2 described above. Conversely, ddPCR analysis of NL63-infected cultures revealed a different outcome: NL63 genome copies in culture supernatants were significantly lower in IFI16KO cells than in TC cells at 144 hpi (*p* = 0.0002) (Fig. 6b), indicating that the impact of IFI16 depletion on HCoV infection is virus-dependent and that NL63 behaves differently from OC43 in our experimental system. We next examined IFI16 subcellular localization upon infection with these viruses and found that both OC43 and NL63 induced cytoplasmic relocalization of IFI16 (Supplementary Fig. 5), suggesting that relocalization is a shared response to HCoV infection despite divergent replication outcomes. Finally, we compared predicted intrinsically disordered regions (IDRs) among HCoV N proteins using IUPred2A (Fig. 6c) and observed that SARS-CoV-2 and OC43 share a similar IDR organization, characterized by a prominent N-terminal disordered region (N-IDR) upstream of the NTD—which, in the case of SARS-CoV-2, has been reported to be crucial for N protein condensation^41^—together with a predicted disordered central linker (LINKER) and a disordered C-terminal region (CTD/tail). In contrast, NL63 lacks a comparable N-terminal IDR, while retaining predicted disorder in the LINKER and C-terminal region (a pattern also observed for HCoV-229E) (Fig. 6c). While these observations are correlative, they highlight differences in N protein architecture that may contribute to the divergent effects of IFI16 depletion observed for SARS-CoV-2 and OC43 versus NL63, and further work will be required to determine whether and how N-terminal disorder influences IFI16-dependent modulation of coronavirus replication.

## Discussion

IFI16 is mostly known for its antiviral activity against DNA viruses replicating in the nucleus, such as *Herpesviridae*^42,43^ and *Papillomaviridae*^17,44^. Upon binding to viral DNA via its two HIN200 domains, IFI16 undergoes cooperative oligomerization mediated by the N-terminal PYRIN (PY) domain leading to antiviral responses such as antiviral cytokine induction and inhibition of viral gene expression^45^. Recent studies have further elucidated this restriction mechanism, revealing the presence of an IDR located between the PY and first HIN domain of IFI16, which is crucial for initiating LLPS nucleated by DNA^28^. More specifically, LLPS regulates IFI16-mediated binding to viral DNA genomes and subsequent cytokine induction during HSV-1 infection, ultimately restricting viral production. However, the role of IFI16 in RNA virus regulation, particularly in CoVs like SARS-CoV-2, remains poorly understood.

**Fig. 7:**
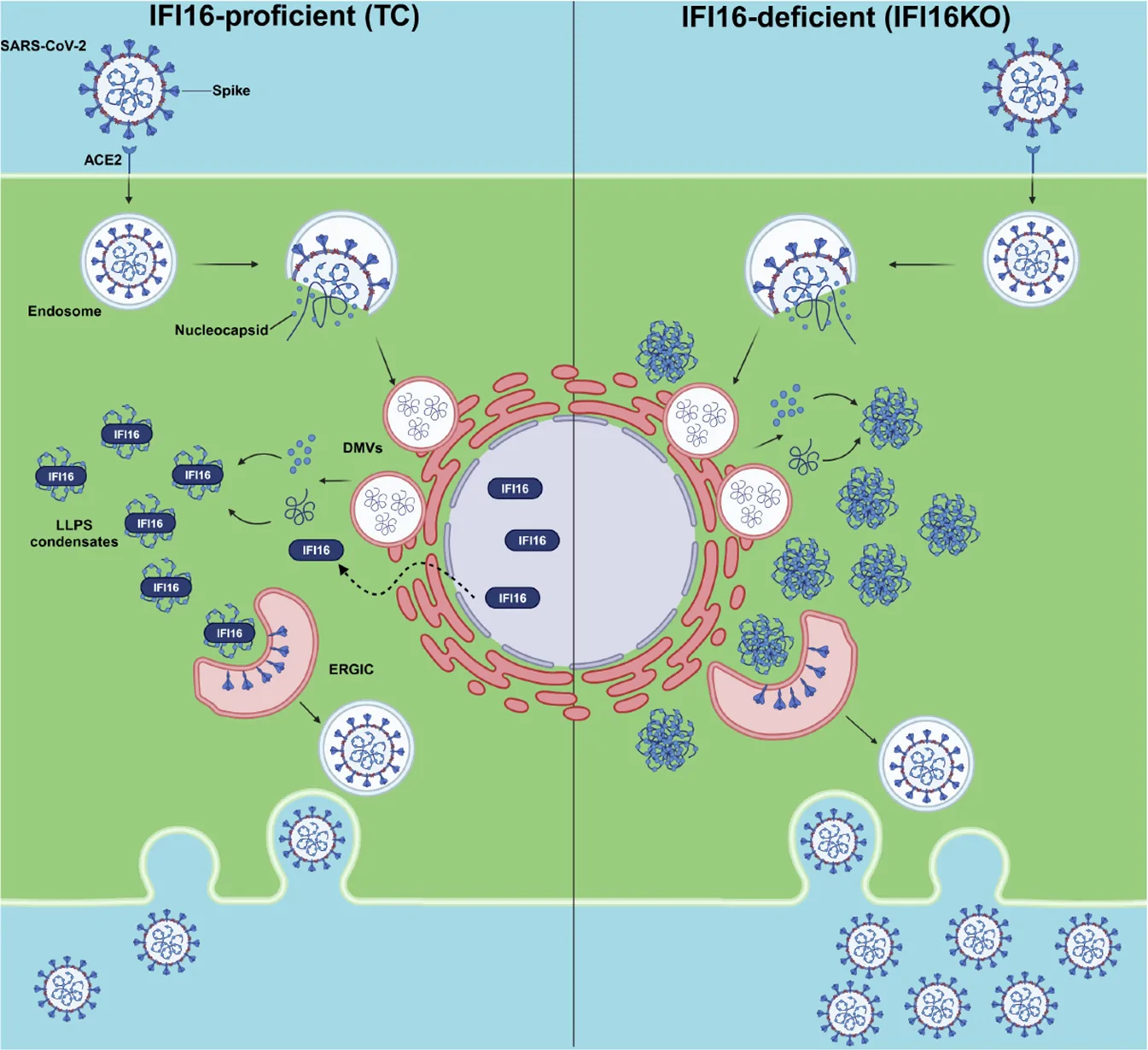
Schematic representation of the viral replication cycle in IFI16-proficient (TC) or IFI16-deficient (IFI16KO) cells. The SARS-CoV-2 spike protein binds to the host receptor angiotensin-converting enzyme 2 (ACE2), promoting viral entry. Once inside the cell, the viral genome is released into the cytosol through uncoating. Viral RNA synthesis and transcription of subgenomic mRNAs occur within double-membrane vesicles (DMVs). The subgenomic mRNAs are then translated into the structural proteins of the virus. Among these, the N protein binds to the newly synthesized viral genomic RNA and undergoes LLPS, promoting viral genome packaging into ribonucleoprotein complexes. Simultaneously, the other viral structural proteins translocate into the endoplasmic reticulum (ER) membranes and pass through the ER-Golgi intermediate compartment (ERGIC), where interaction with N-encapsidated, newly produced genomic RNA results in budding into the lumen of the secretory vesicular compartment. Finally, virions are secreted from the infected cell through exocytosis. In IFI16-proficient cells (TC; left panel), after infection, IFI16 translocates from the nucleus to the cytoplasm, where it binds to the N protein and interferes with the LLPS phenomenon. Conversely, in IFI16-deficient cells (IFI16KO; right panel), the viral genome is efficiently packaged into ribonucleoprotein complexes. Due to the absence of IFI16 interference in N/vRNA LLPS, N condensates are larger, and IFI16-deficient cells release a higher number of virions compared to IFI16-proficient cells. Created with BioRender.com licensed under CC BY 4.0. https://BioRender.com/i79b753.

This study addresses this gap by providing the first evidence of the involvement of IFI16 in controlling SARS-CoV-2 replication through disruption of vRNA-induced LLPS of the N protein. This antiviral activity is dependent on the translocation of IFI16 from the nucleus to the cytoplasm, where it binds to both vRNA and the N protein (Fig. 7). Using biochemical and cellular approaches, including high-resolution fluorescent microscopy, PLA, SPR, immunoprecipitation, and cross-linking RNA immunoprecipitation, we show for the first time that IFI16 forms a cytoplasmic complex with genomic SARS-CoV-2 RNA and the viral N protein. Importantly, in our experimental system, this interaction was not accompanied by a marked induction of IFN-β or representative ISGs. Thus, while we cannot exclude more subtle effects, our data suggest that IFI16-mediated restriction occurs without a strong type I IFN signature under these conditions.

An important finding of this work is that the N condensates in SARS-CoV-2-infected IFI16KO cells were clearly detectable as early as 24 hpi, displaying perinuclear localization with significant overlap with the envelope protein S, indicating that viral particle formation occurred earlier and more extensively in these cells compared to similarly infected IFI16-proficient cells. Notably, in IFI16-proficient cells, the N puncta were rare and barely detectable at the early time points due to the diffuse cytoplasmic localization of the N protein. Furthermore, the overlap with the S protein was only detectable at later time points pi (e.g., 48 hpi), and the size of N condensates never reached the dimensions observed in IFI16-deficient cells, where they were significantly larger. Thus, these findings suggest that the absence of IFI16 fosters a cellular environment that promotes a more efficient and rapid viral replication cycle. Although most experiments were performed in the LLC-MK2 cell line, selected for its high permissiveness to SARS-CoV-2 and ability to support productive infection by all tested coronaviruses, the restrictive effect of IFI16 was also reproduced in the human Calu-3 cells, in which IFI16 silencing increased viral replication. Overall, our data support a role for cytoplasmic IFI16 as a regulator of coronavirus replication.

Recent data indicate that the N protein may form nucleocapsid complexes with viral genomes via LLPS^7,11,37^. These nucleocapsids then transit through the ER-to-Golgi intermediate compartment (ERGIC) for interaction with structural proteins, such as the S protein, to assemble mature virions^46^. Based on this, we propose that the increased double-labeled condensates observed in IFI16KO cells, predominantly in the perinuclear region, represent virus assembly sites typically located in the Golgi apparatus (Fig. 7), and that their formation could be promoted by enhanced N-vRNA interactions and phase separation-driven compartmentalization in the absence of IFI16’s inhibitory effect. This interpretation is consistent with the significantly higher viral yield observed in culture supernatants from IFI16-deficient cells, along with earlier expression of S protein.

The first evidence of IFI16 acting as an antiviral RBP was reported by Jiang et al. 2021, who demonstrated that IFI16 inhibits IAV replication by inducing type I IFN production through RIG-I activation and direct vRNA binding^26^. Unlike IAV, in our experimental model IFI16 was not associated with a measurable modulation of IFN-β production or representative ISG expression during SARS-CoV-2 infection, suggesting that IFI16-mediated restriction can occur without a prominent type I IFN signature under these conditions. Similarly, IFI16 restricts the replication of CHIKV—a single-stranded positive-sense RNA virus—by binding to viral RNA, independently of IFN signaling or DNA-binding transcriptional activity^27^. These findings suggest that the antiviral effects of IFI16 extend beyond innate immune response modulation, involving direct interaction with viral RNA in its cytoplasmic localization.

IFI16 was previously identified as an interaction partner of the SARS-CoV-2 N protein in HEK293T and Calu-3 cells using affinity purification and mass spectrometry^47^. We now demonstrate that IFI16 protein disrupts LLPS of SARS-CoV-2 N protein with genomic vRNA, which in turn generates higher-order protein/RNA complexes needed for efficient viral replication. Consequently, the lack of IFI16 protein favors the formation of membraneless organelles that enhance viral replication.

When we tested low-pathogenic HCoVs, OC43 mirrored the phenotype observed for SARS-CoV-2, with increased viral genome copies in IFI16KO supernatants, supporting a conserved restrictive role of IFI16 against these betacoronaviruses. In contrast, NL63 genome copies were reduced upon IFI16 depletion, underscoring that IFI16-dependent modulation of replication is virus-specific. Interestingly, this divergence parallels differences in predicted N-protein intrinsically disordered regions, as SARS-CoV-2 and OC43 share a prominent N-terminal IDR that is not evident in NL63, suggesting that N-protein architecture may contribute to IFI16-sensitive replication outcomes.

In summary, our study provides new insights into the role of IFI16 in modulating CoV replication. We demonstrate that IFI16 acts as an antiviral factor for SARS-CoV-2 and OC43, whereas it promotes NL63 replication. Moreover, our findings emphasize the biological relevance of LLPS-based processes during SARS-CoV-2 replication and demonstrate how host proteins, particularly interferon-inducible factors like IFI16, can interfere with these mechanisms to regulate viral replication.

## Methods

### Biosafety statements and facility

All experiments with live NL63 and OC43 were performed in a biosafety level 2 (BSL2) facility at the Università del Piemonte Orientale, Novara, Italy. Experiments involving live SARS-CoV-2 were performed in biosafety levels 3 (BSL3) facility at the Università degli Studi di Milano, Milan, Italy. The standard operating procedures for both BSL2 and BSL3 facilities were approved by relevant authorities in Italy. All personnel underwent comprehensive training prior to beginning work in the BSL2 and BSL3 facilities.

### Cells and viruses

Rhesus monkey kidney epithelial cells (LLC-MK2, ATCC: CCL-7), African green monkey cells (Vero E6, ATCC: CRL-1586), human colorectal cancer cells (Caco-2,ATCC: HTB-37), human lung fibroblasts (MRC-5, IRCCS Ospedale Policlinico San Martino) and human lung adenocarcinoma cells (Calu-3, ATCC: HTB-55) were cultured in high glucose Dulbecco’s modified Eagle’s medium (DMEM), supplemented with 10% fetal bovine serum (FBS) and 1% penicillin-streptomycin. Cells were maintained at 37 °C with 5% CO_2_.

The HCoV strain NL63 (NR-470, also referred to as Amsterdam I, Bei Resources) and the HCoV strain OC43 (ATCC® VR-1558) were kindly provided by Lucia Nencioni (Università della Sapienza, Rome, Italy) and David Lembo (Department of Clinical and Biological Sciences, University of Turin, Turin, Italy), respectively. HCoV-NL63 and HCoV-OC43 were propagated and maintained in our laboratory using Caco-2 and MRC-5 cells, respectively. Both viruses were titrated as plaque-forming units by immunofluorescence staining of the nucleocapsid protein.

All experiments involving SARS-CoV-2 were conducted in collaboration with Serena Delbue (University of Milan). SARS-CoV-2 was isolated from a nasal-pharyngeal swab positive for SARS-CoV-2. The isolated SARS-CoV-2 strain belongs to the B.1 lineage, carrying the characteristic spike mutation D614G, which corresponds to the predominant European lineage linked to the Northern Italian outbreak in early 2020. The complete nucleotide sequence has been deposited in GenBank and GISAID (accession Nos. MT748758.1 and EPI_ISL 584051, respectively). SARS-CoV-2 was propagated and maintained in the Laboratory of Molecular Virology at the University of Milan using Vero E6 cells. SARS-CoV-2 was titrated as plaque-forming units by plaque assay.

### Generation of IFI16KO LLC-MK2 cells

LLC-MK2 IFI16KO and TC cells were generated using CRISPR/Cas9 technology, as previously described^48,49^. Briefly, vesicular stomatitis virus G (VSV-G)-pseudotyped lenti-CRISPR virions were produced by transfecting HEK293T cells with the following plasmids: CRISPR/Cas9 vector and Virapower lentiviral packaging mix (Invitrogen). Viral supernatants were collected after 72 h and used to transduce LLC-MK2 cells by infection in the presence of 10 µg /ml polybrene. Transduced cells were selected with puromycin (10 µg/ml) at 2 days post-transduction. After 2 weeks a single cell suspension culture was established using limiting dilution. After 3 weeks individual clones were subjected to western blotting to confirm absence of the targeted gene products.

### Quantitative real-time PCR

qRT-PCR was performed using a CFX96 Real-Time PCR Detection System (Bio-Rad Laboratories), as previously described^50^. Amplification of subgenomic *N*, *E*, and *S* genes was carried out using sg leader forward primers and the *N*, *E*, and *S* reverse primers listed in Table S1 and the following reaction conditions: 2 min at 95 °C, 40 cycles of 5 s at 95 °C, 10 s at 55 °C, 20 s at 72 °C, followed by 5 min at 72 °C and 10 min at 4 °C. Total RNA was extracted using TRI Reagent (Sigma-Aldrich), and 1 μg was retrotranscribed using an iScript cDNA Synthesis Kit (Bio-Rad Laboratories). Reverse-transcribed cDNAs were amplified in duplicate using SsoAdvanced Universal SYBR Green Supermix (Bio-Rad Laboratories). *GAPDH* was used as housekeeping gene for normalization of cDNA levels. The relative normalized expression after stimulation was calculated as fold change over control using the formula = 2 -Δ(ΔCT) where ΔCT = CTtarget—CTGAPDH and Δ(ΔCT) = ΔCTstimulated – ΔCTcontrol.

The primers used are listed in Table S1.

### Droplet digital PCR

ddPCR was performed using the one-step RT-ddPCR advanced kit for probes (Bio-Rad Laboratories) according to the manufacturer’s instructions on the Bio-Rad QX200 (Bio-Rad Laboratories). PCR cycling conditions were as follows: 60 min reverse transcription at 50 °C, 10 min enzyme activation at 95 °C, 30 s denaturation at 94 °C (40 cycles or 45 cycles, for SARS-CoV-2 and NL63, respectively), 1 min annealing/extension cycle at 55 °C (40 cycle for SARS-CoV-2 and OC43 or 45 cycles for NL63, respectively; ramp rate of 2–3 °C/s), 10 min enzyme deactivation at 98 °C and a 30-min hold at 4 °C. Positive and negative droplet readings were performed using a Bio-Rad Droplet Reader (Bio-Rad Laboratories).

The primers used are listed in Table S2.

### Plaque assay

Vero cells were seeded at a density of 7.5 × 10⁵ cells per well in 6-well plates and cultured in complete DMEM medium (Euroclone) composed of 10% fetal bovine serum (FBS) (Euroclone) and 1× penicillin/streptomycin (Euroclone) until confluence. SARS-CoV-2-infected cell mediums were 10-fold serially diluted in complete DMEM medium and used to infect the cell monolayers. After 2 h of adsorption at 37 °C with 5% CO_2_, the inoculum was removed, each well was washed twice with 2 mL of PBS (Euroclone) and cells were overlaid with 3 ml of complete DMEM medium containing 0.3% (m/V) agarose and incubated for 48 h at 37 °C with 5% CO_2_. Cells were then fixed with 4% formaldehyde solution (Sigma-Aldrich) for 1 h at room temperature. The agarose overlay was then removed, each well was washed twice with 2 mL of PBS (Euroclone), and cells were stained with methylene blue (0.4 g/L) for 15 min at room temperature. Each well was washed three times with 2 mL of water and allowed to dry overnight. Viral plaques were counted, and titers were expressed as plaque-forming units per milliliter (PFU/mL).

### Immunoblotting

Whole-cell protein extracts (15 µg) were prepared, separated by SDS-PAGE, transferred to nitrocellulose membrane, and subjected to immunoblot analysis as previously described^51^. The following primary antibodies were used: rabbit polyclonal antibodies (pAbs) anti-IFI16 (in-house made^52^, 1:1000), rabbit mAbs anti-SARS-CoV-2 N (40143-R001; SinoBiological, 1:2000), mouse mAbs anti-SARS-CoV-2 S (GTX632604; GeneTex, 1:500), and anti-GAPDH (60004-1-Ig; Proteintech, 1:10000). Immunocomplexes were detected using sheep anti-mouse or donkey anti-rabbit immunoglobulin antibodies conjugated to horseradish peroxidase (HRP) (GE Healthcare Europe GmbH) and visualized by enhanced chemiluminescence (Super Signal West Pico; ThermoFisher Scientific) using the ChemiDoc Touch Imaging System (Bio-Rad Laboratories).

### IFI16 silencing in Calu-3 cells

Calu-3 cells were seeded 24 h prior to transfection to reach ~60–70% confluence at the time of treatment. IFI16 expression was silenced using small interfering RNA (siRNA) targeting human IFI16 (IFI-16 siRNA (h): sc-35633, Santa Cruz Biotechnology) and a non-targeting control siRNA as negative control (siRNA negative control (HY-RS06552), MedChemExpress). Transfections were performed using Lipofectamine 3000 Transfection Kit (L3000-015, Thermo Fisher Scientific) according to the manufacturer’s instructions. siRNAs were used at a final concentration of 50 nM.

Cells were incubated for 48 h post-transfection before SARS-CoV-2 infection. Silencing efficiency was verified by immunoblotting and immunofluorescence.

### Immunoprecipitation and RNA-immunoprecipitation assays

Immunoprecipitation (IP) of IFI16 with interacting proteins was performed using the Dynabeads Protein G Immunoprecipitation Kit (ThermoFisher Scientific, Invitrogen), according to the manufacturer’s instructions, with minor modifications. Briefly, cytoplasmic cell extracts were obtained with the NE-PER Nuclear and Cytoplasmic Extraction Reagents kit (ThermoFisher Scientific, Invitrogen) according to the manufacturer’s instructions. Fifteen μg of total cell extracts were kept as the input control, while 300 μg of cytoplasmic cell extracts were incubated for 2 h at 4 °C with 3 μg of anti-IFI16 (in-house made^52^) or anti-IgG (C15410206; Diagenode) pAbs, previously conjugated with magnetic beads. The resulting complexes were then washed, eluted, denatured, and subjected to immunoblotting as described above. Immunocomplexes were detected using TidyBlot Western Blot Detection Reagent:HRP (STAR209PA; Bio-Rad Laboratories) and visualized by enhanced chemiluminescence (Super Signal West Pico; ThermoFisher Scientific) using the ChemiDoc Touch Imaging System (Bio-Rad Laboratories). For RNase-treated cell extracts, Ambion RNase A (ThermoFisher Scientific, Invitrogen) was added at a concentration of 0.005 μg/μl and incubated for 15 min at 37 °C.

IFI16 RIP was performed using the same protocol as that employed for IP, with minor modifications, as 7 mg of cytoplasmic cell extracts were incubated for 2 h at 4 °C with 10 μg of anti-IFI16 (in-house made^52^) or anti-IgG (C15410206; Diagenode) pAbs, previously conjugated with magnetic beads. Immunoprecipitated RNA was extracted using the PureLink RNA Mini Kit (ThermoFisher Scientific, Invitrogen) according to the manufacturer’s instructions and subjected to RT-qPCR as described above.

The primers used are listed in Supplementary Table 1.

### Immunofluorescence

At the indicated time points, cells were fixed with 4% paraformaldehyde (PFA) and then incubated O/N at 4 °C using appropriate dilutions of primary antibody in a humidified chamber, followed by secondary labeled antibody for 1 h. DAPI (4′,6′-diamodino-2phenylindole) (ThermoFisher Scientific, Invitrogen) was used to counterstain the nuclei. The following primary and secondary antibodies were used: mouse mAbs anti-IFI16 (sc-8023; Santa Cruz, 1:100), anti-dsRNA (MABE1134; Sigma-Aldrich; 1:100), and anti-SARS-CoV-2 S (GTX632604; GeneTex, 1:500), rabbit mAb anti-SARS-CoV-2 N (40143-R001; SinoBiological, 1:2000), goat anti-mouse IgG-Alexa Fluor 488 (A11001; Thermo Fisher Scientific), goat anti-mouse IgG-Alexa Fluor 568 (A11004; Thermo Fisher Scientific), goat anti-rabbit IgG-Alexa Fluor 488 (A11008; Thermo Fisher Scientific), and goat anti-rabbit IgG-Alexa Fluor 568 (A11011; Thermo Fisher Scientific). The coverslips were mounted with SlowFade Gold antifade reagent mounting media (ThermoFisher Scientific, Invitrogen), and cells visualized using a Leica SP8 lightning confocal microscope (Leica Microsystems). The percentage of infected cells expressing N or dsRNA for each cell line was normalized to the total number of DAPI-positive cells. Image analysis was carried out using the LAS X software (Leica Microsystems), and values were expressed as mean ± SD (error bars). Differential interference contrast (DIC) and fluorescence images for LLPS experiments were visualized using a Leica DM6000 B microscope (Leica Microsystems) and processed with FIJI software (version 1). For fluorescence imaging, N and IFI16 were labeled with Alexa Fluor 488 or Alexa Fluor 594 microscale protein labeling kits (Thermo Fisher Scientific).

### Super-resolution (Airyscan) microscopy and image analysis

Super-resolution microscopy (spatial resolution: 120–140 nm) was performed using a Zeiss LSM 900 with Airyscan detector (Carl Zeiss), equipped with GaAsP detectors (Gallium:Arsenide:Phosphide). Samples were viewed with a 63x Apochromat NA = 1.4 oil-immersion objective. A pixel size ≤0.03 mm was adopted to ensure full Nyquist spatial sampling without loss of spatial resolution. The pinhole size was set to 1 airy unit (AU). Pixel dwell time was adjusted to 1.52 μs and four averages were collected in each channel. The acquisition channels were set as follows:

- Blue (Hoechst 33342, to visualize chromatin): *λ*ex = 405 *λ*em = 420–500 nm

- Green (Alexa Fluor 488, to visualize N protein of SARS-CoV-2): *λ*ex = 488, *λ*em = 500–560 nm

- Red (Alexa Fluor 568, to visualize S protein of SARS-CoV-2): *λ*ex = 561, *λ*em = 580–600 nm

Images were processed using the open-source software Fiji (NIH, Bethesda). Colocalization analysis was carried out according to a previously published procedure^53^. First, the green and red Airyscan images of the same field were background-subtracted by the internal “background-subtraction” routine of FiJi (50 pixel rolling ball). Next, the two images were cropped to the same region of interest (ROI) to minimize spurious co-localization signals from regions outside the cell cytoplasm. Finally, the Pearson’s colocalization coefficient (R) was determined with the JACoP colocalization plugin^54^, according to the Costes’ method^55^. Grayscale histogram entropy was determined by applying the histogram statistics texture analysis plugin (https://github.com/IES-HelmholtzZentrumMunchen/imagej-histogram-statistical-texture-analysis) of ImageJ/FiJi to an ROI enclosing the cell cytoplasm in the green channel image. The plugin directly calculated the entropy value of the ROI, along with other histogram metrics. Granulometry analysis was performed using the IJGranulometry (mode: grayscale granulometry in radius) plugin (https://github.com/ijtools/ijGranulometry) of ImageJ/FiJi to the green channel image. Analysis parameters were as follows: operation = closing, element = square, radius max = 50 pixel, step = 1 pixel, spatial calibration = 0.033 mm. The plugin yielded the granulometry distribution curve G (d), where d is the diameter (in mm) of the probing square, with d ranging from 0.099 to 3.268 mm in 49 discrete steps. The mean granulometric diameter $\hat{d}$ was obtained as follows:

$$\hat{d}={\sum }_{0.099}^{3.268}d\bullet G(d)/{\sum }_{0.099}^{3.268}G(d)$$

### Proximity ligation assay

At the indicated time points, cells were fixed with 4% paraformaldehyde, and the DuoLink PLA kit (Sigma-Aldrich) was used according to the manufacturer’s instructions. Primary antibodies were incubated overnight (O/N) at 4 °C in a humidified chamber at appropriate dilutions. DAPI was used to counterstain the nuclei. The following pAbs were used: mouse mAbs anti-IFI16 (sc-8023; Santa Cruz, 1:100) and rabbit mAb anti-SARS-CoV-2 N (40143-R001; SinoBiological, 1:2000). Coverslips were mounted using SlowFade Gold antifade reagent mounting media (ThermoFisher Scientific, Invitrogen), and cells visualized using an AxioScan 7 Microscope Slide Scanner (Carl Zeiss). The number of PLA spots was counted using FIJI software and normalized to the total number of DAPI-positive cells. Values were expressed as mean ± SD.

### Surface plasmon resonance

A Biacore X100 (GE Healthcare) instrument was used for real-time binding interaction experiments. Recombinant IFI16 obtained as previously described^56^ was covalently immobilized onto the surface of sensor CM5 chips (cat # BR100012, GE Healthcare) via amine coupling. IFI16 was diluted to 25 μg/ml in 10 mM sodium acetate at pH 4.0. Recombinant protein was injected on CM5 chip at a flow rate of 10 μl/min, upon activation of the carboxyl groups on the sensor surface with 7-min injection of a mixture of 0.2 M EDC and 0.05 M NHS. The remaining esters were blocked with 7 min injection of ethanolamine. The appropriate ligand density (RL) on the chip was calculated according to the following equation: RL = (ligand MW/ analyte MW) × Rmax × (1/Sm), where Rmax is the maximum binding signal and Sm corresponds to the binding stoichiometry. Recombinant IFI16 (in-house made) and analytes (N of SARS-CoV-2, Z03488; GenScript) had molecular weights (MW) of 90 kDa and 46 kDa, respectively. The target capture level of IFI16 was of 1926.6 response units (RUs). The other flow cell was used as a reference and was blocked immediately after activation.

Increasing concentrations of recombinant SARS-CoV-2 N were flowed over the CM5 sensor chip coated with IFI16 at a flow rate of 30 μl/min at 25 °C, with an association time of 30 s and a dissociation phase of 180 s. A single regeneration step with 50 mM NaOH was performed following each analytic cycle. All the analytes tested were diluted in the PBSP+ buffer (GE Healthcare). The KDs were evaluated using the BIAcore evaluation software (GE Healthcare), and the reliability of the kinetic constants calculated by assuming a 1:1 binding model supported by the quality assessment indicators.

### Phase separation assay

Phase separation experiments of N proteins (30 μM or 50 μM) with polyU (0.05–1 μM) and addition of IFI16 protein (1–3 μM) were performed in 20 mM NaP buffer, pH 7.5, at room temperature (RT).

### Turbidity measurements

Absorbance values of N protein samples at different polyU and IFI16 concentrations were determined at a wavelength of 350 nm using a NanoDrop spectrophotometer (ThermoFisher Scientific). Samples were prepared by mixing N and IFI16, and polyU was added last, all at RT. For IFI16 dissolution experiments, 1 μl of IFI16/buffer was added after droplets were formed by polyU. Average turbidity values were calculated from 9 samples, comprising three freshly prepared, independent samples and three replicates per sample.

### Protein purification

N and IFI16 proteins were purified according to the protocol previously published^57^. Briefly, proteins with a HIS-tag were expressed in *E. coli* in minimal medium and purified by a combination of cation exchange and Ni sepharose column. The HIS-tag was cut by TEV or EK proteases, respectively. Subsequently, the tag was removed by another run on Ni sepharose column. In the final step, the proteins were purified by gel filtration in 20 mM NaP (sodium phosphate) buffer, pH 7.6.

### Statistical analysis

All statistical tests were performed using Graph-Pad Prism version 7.00 for Windows (GraphPad Software). The data are stated as mean ± standard deviation (SD). For comparisons consisting of two or more groups, means were compared using unpaired Student’s *t*-test, two-tailed Student’s *t*-test, Mann–Whitney test or two-way ANOVA. Differences in *p*-value < 0.05 were considered statistically significant.

### Reporting summary

Further information on research design is available in the Nature Portfolio Reporting Summary linked to this article.

## Supplementary information

Supplementary Information

Description of Additional Supplementary Materials

Supplementary data 1-6

Reporting Summary

Transparent Peer Review file

## Data Availability

Numerical data from the analyses are available in the Supplementary Materials (Supplementary Data 1–6). Supplementary Figs. S6–S9 show the original western blots corresponding to Figs. 1c, 4e, f and S1. All other data are available from the corresponding author upon reasonable request.
