# Supplementary material for: IFI16 restricts SARS-CoV-2 replication by disrupting nucleocapsid-driven phase separation
Source: Commun Biol. 2026 Jun 1;9:1255. doi: 10.1038/s42003-026-10363-0 (PMC13614959; doi:10.1038/s42003-026-10363-0)

Supplementary Table 1. Primers used for quantitative real-time PCR

	Gene	Primer
**SARS-CoV-2**	*ORF1ab* forward	GTGARATGGTCATGTGTGGCGG
	ORF1ab reverse	CARATGTTAAASACACTATTAGCATA
	Sg leader N and *E* forward	CGATCTCTTGTAGATCTGTTCTC
	N reverse	TCTGGTTACTGCCAGTTGAATCTG
	E reverse	ATATTGCAGCAGTACGCACACA
	Sg leader S forward	CCAACTTTCGATCTCTTGTAG
	S reverse	AGAACAAGTCCTGAGTTGAATG
**Housekeeping**	*GAPDH* forward	TCACCACCATGGAGAAGGC
	GAPDH reverse	GCTAAGCAGTTGGTGGTGCA
**Type I IFNs**	*IFN*β forward	GTCTCCTCCAAATTGCTCTC
	*IFN*β reverse	ACAGGAGCTTCTGACACTGA
**ISGs**	*Mx1* forward	AGGACCATCGGAATCTTGAC
	Mx1 reverse	TCAGGTGGAACACGAGGTTC
	IFIT-1 forward	TTGCCTGGATGTATTACCAC
	IFIT-1 reverse	GCTTCTTGCAAATGTTCTCC

Supplementary Table 2. Primers used for droplet digital PCR

	Gene	Primer
**NL63**	*N* forward	AGGACCTTAAATTCAGACAACGTTCT
	N reverse	GATTACGTTTGCGATTACCAAGACT
	N probe	CCAAACAT-BHQ1
**SARS-CoV-2**	*N* forward	GAC CCC AAA ATC AGC GAA AT
	N reverse	CGATCTCTTGTAGATCTGTTCTC
	N probe	FAM-ACCCCGCATTACGTTTGGTGGACC-BHQ1
**OC43**	*N* forward	AGCAACCAGGCTGATGTCAATACC
	N reverse	AGCAGACCTTCCTGAGCCTTCAAT
	N probe	FAM-TGACATTGTCGATCGGGACCCAAGTA-TAMRA

Supplementary Figure S1

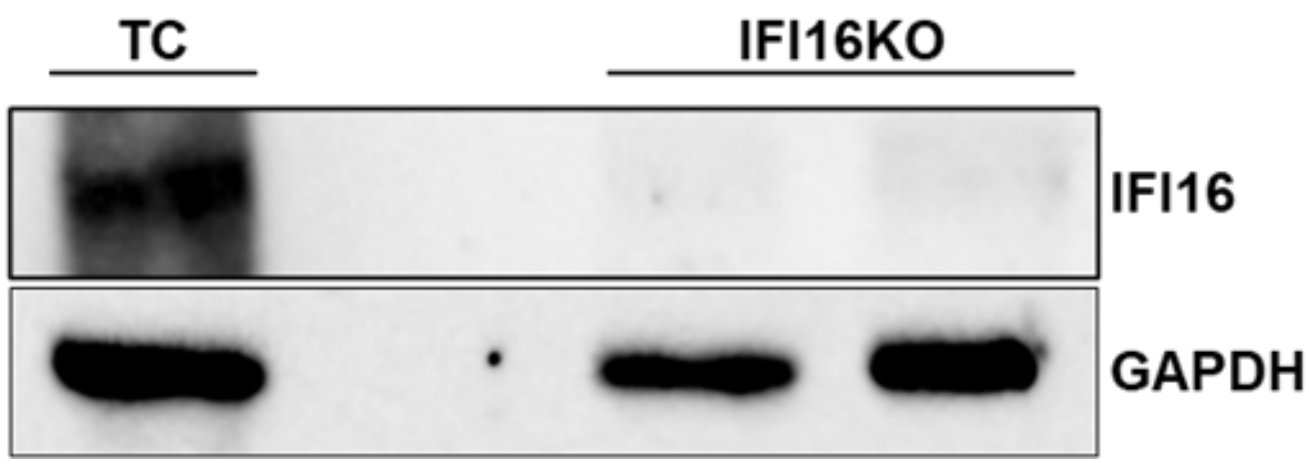

Figure S1. **Knock-out of IFI16 in LLC-MK2 cells.** Total cell extracts from TC and two independent clones of IFI16KO cells were subjected to immunoblot analysis using an anti-IFI16 polyclonal antibody. GAPDH immunodetection was used as a loading control. One representative Western blot from three independent experiments is shown (Original western blot shown in Figure S9).

Supplementary Figure S2

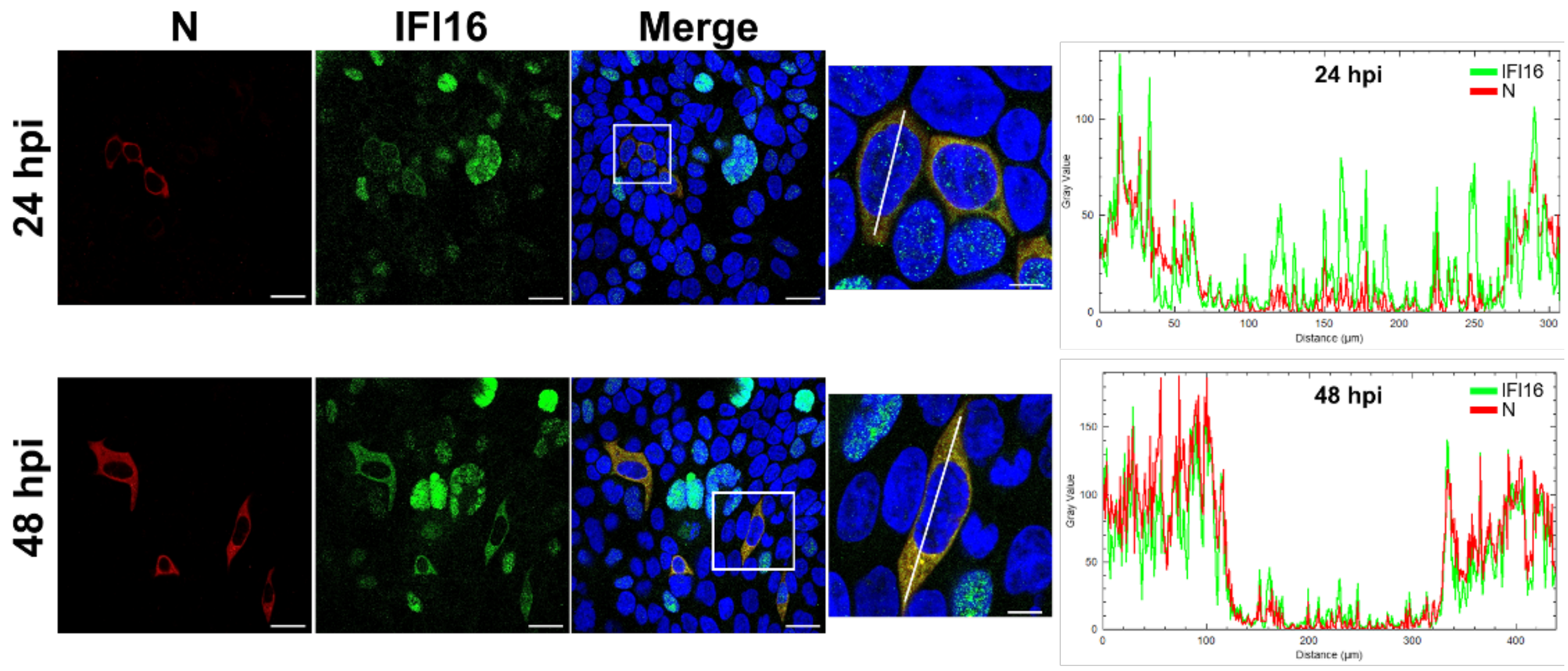

Supplementary Figure S2. IFI16 protein translocates to the cytoplasm upon HCoV infection in Calu-3 cells. Calu-3 cells were infected with SARS-CoV-2 at an MOI of 0.5. At the indicated hpi cells were processed for immunofluorescence analysis for N (red) and IFI16 (green). Nuclei were visualized by DAPI (blue). Images were captured by confocal microscopy and are representative of three independent experiments. Scale bars: 25 µm. The images in the last column correspond to the magnified areas highlighted by a white square in the merged image. Scale bars: 10 µm. The fluorescence intensity profiles of IFI16 (green) and N (red) across different areas of the magnified cells, as indicated by the white line, are plotted. The graphs show the distribution and colocalization of N and IFI16 proteins as determined by ImageJ/FiJi.

Supplementary Figure S3

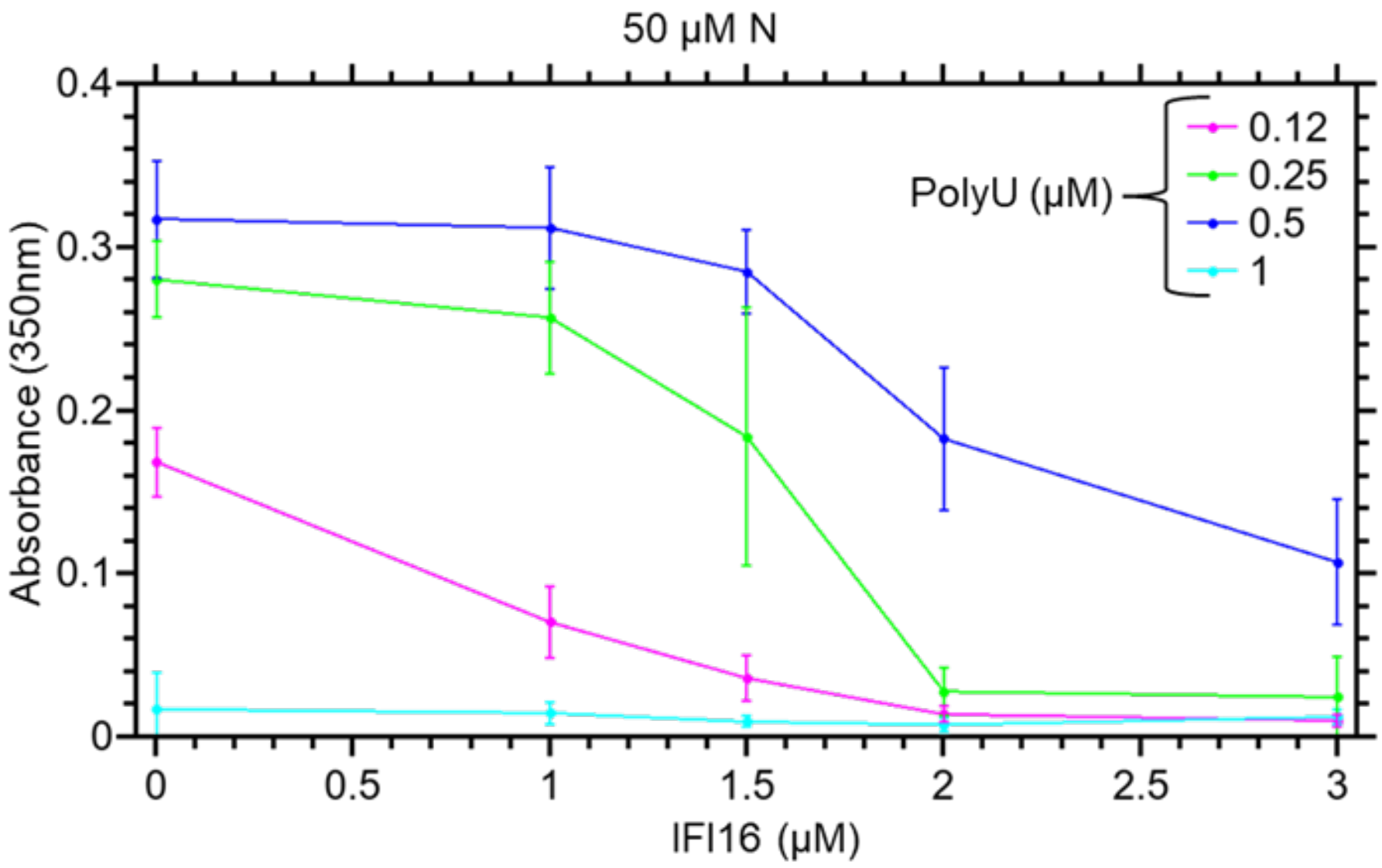

Figure S3. Influence of IFI16 protein concentration on the formation of N/RNA condensates. Turbidity measurements of N protein (50 µM) in the presence of increasing concentrations of polyU (0.12-1 µM) and IFI16 (1-3 µM), in 20 mM NaP buffer, pH 7.5. Data are presented as mean ± SD (n = 3 independent samples and 3 measurements per samples).

Supplementary Figure S4

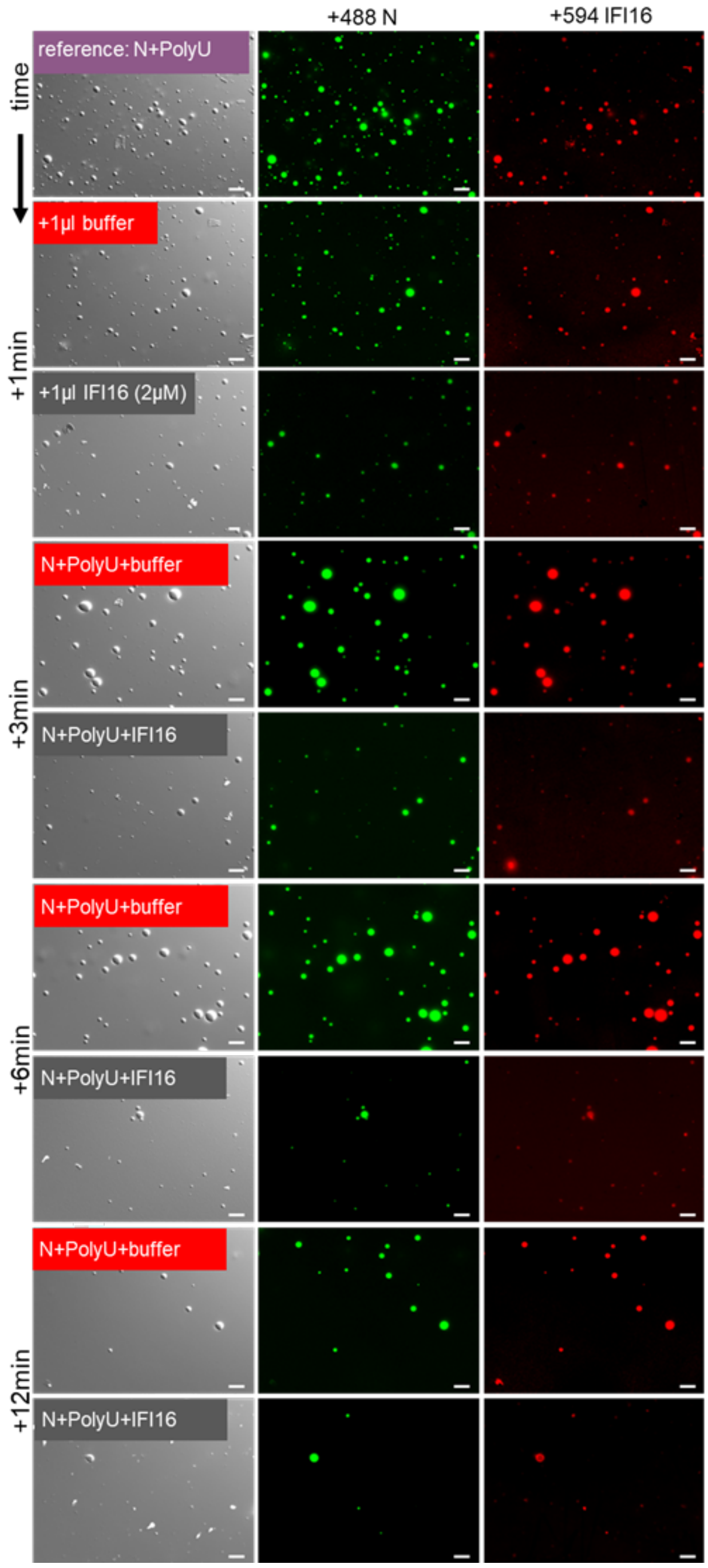

Figure S4. Time-dependent dissolution of N/RNA condensates in the presence of IFI16. DIC and fluorescence images showing the dissolution effect of IFI16 (grey boxed titles) on N /RNA condensates (purple boxed title) compared to the control sample (red boxed titles), where 1 µl of buffer was added to account for the dilution effect due to the addition of 1 µl of IFI16. For fluorescence imaging, samples were mixed with Alexa 488 labeled N protein (green) and Alexa 594 labeled IFI16 protein (red). Scale bars: 10 µM.

Supplementary Figure S5

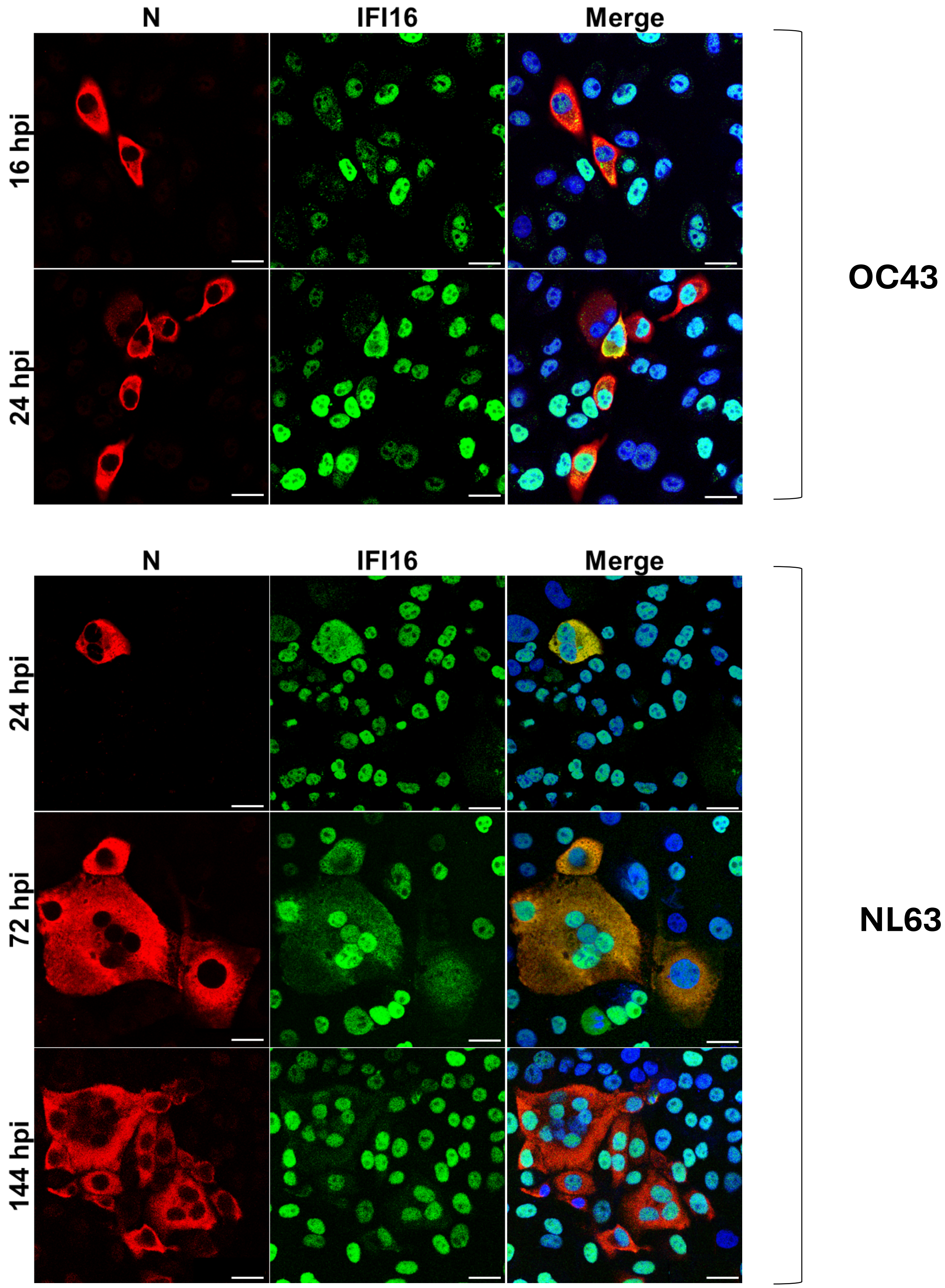

Figure S5. The IFI16 protein translocates to the cytoplasm upon OC43 and NL63 infection. TC cells were infected with OC43 (upper panel) or NL63 (lower panel) at an MOI of 0.01 and 1, respectively, and processed for immunofluorescence analysis for IFI16 (green) and N (red). Nuclei were visualized by DAPI (blue). Images were captured by confocal microscopy and are representative of six different fields from three independent experiments. Scale bars: 25 μm.

Supplementary Figure S6. Original western blot of Figure 1c

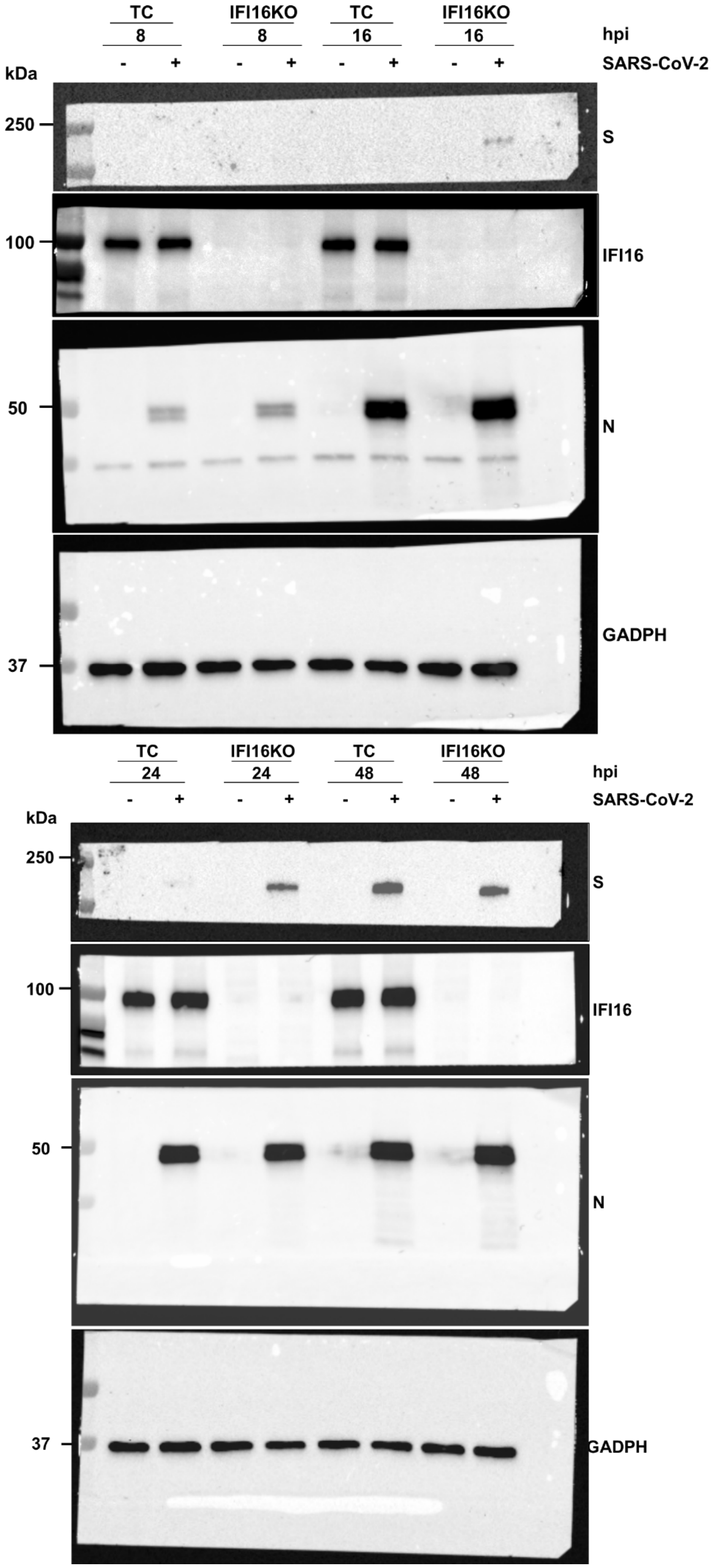

Supplementary Figure S7. Original western blot of Figure 4e

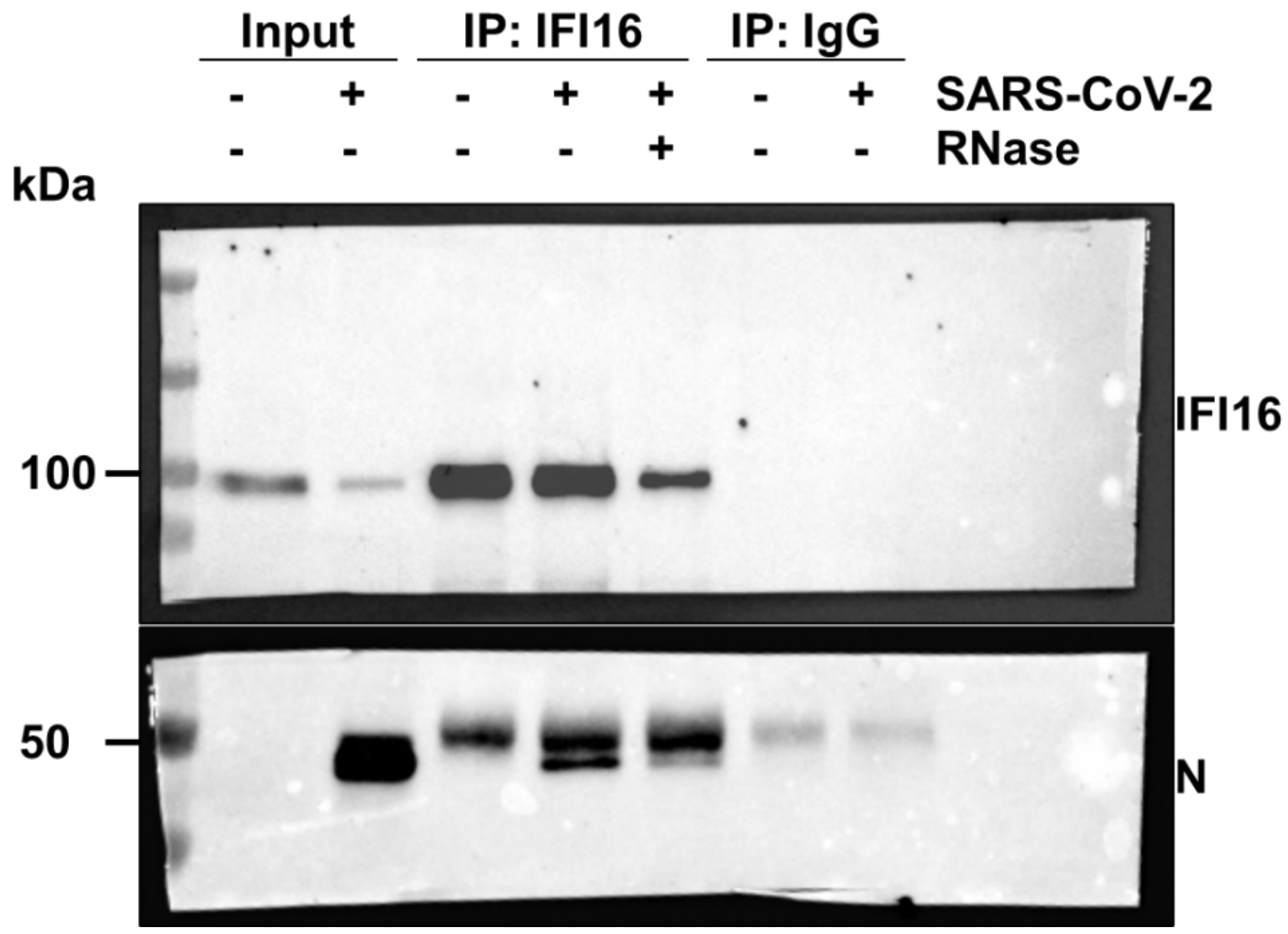

Supplementary Figure S8. Original western blot of Figure 4f

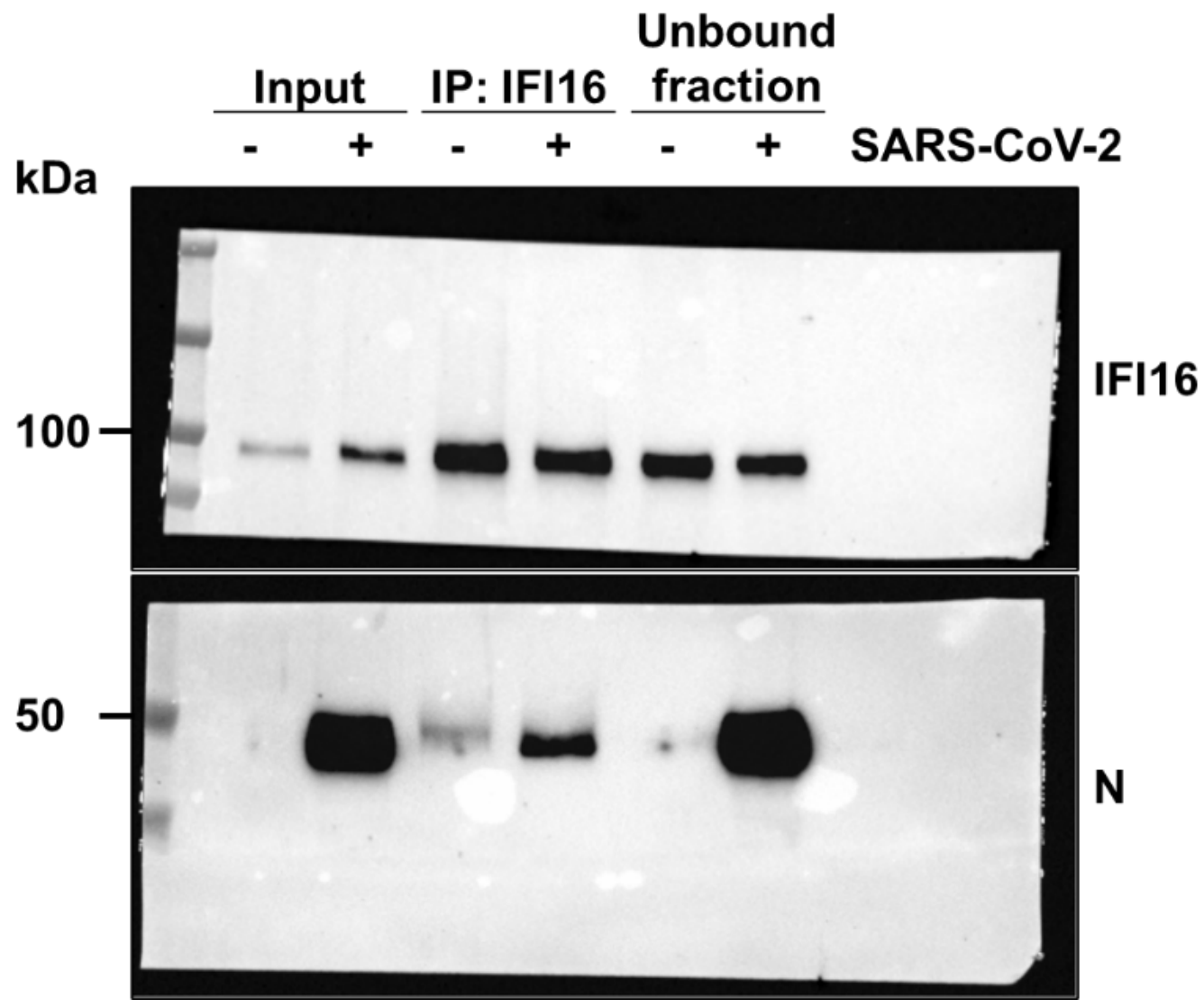

Supplementary Figure S9. Original western blot of Figure S1.

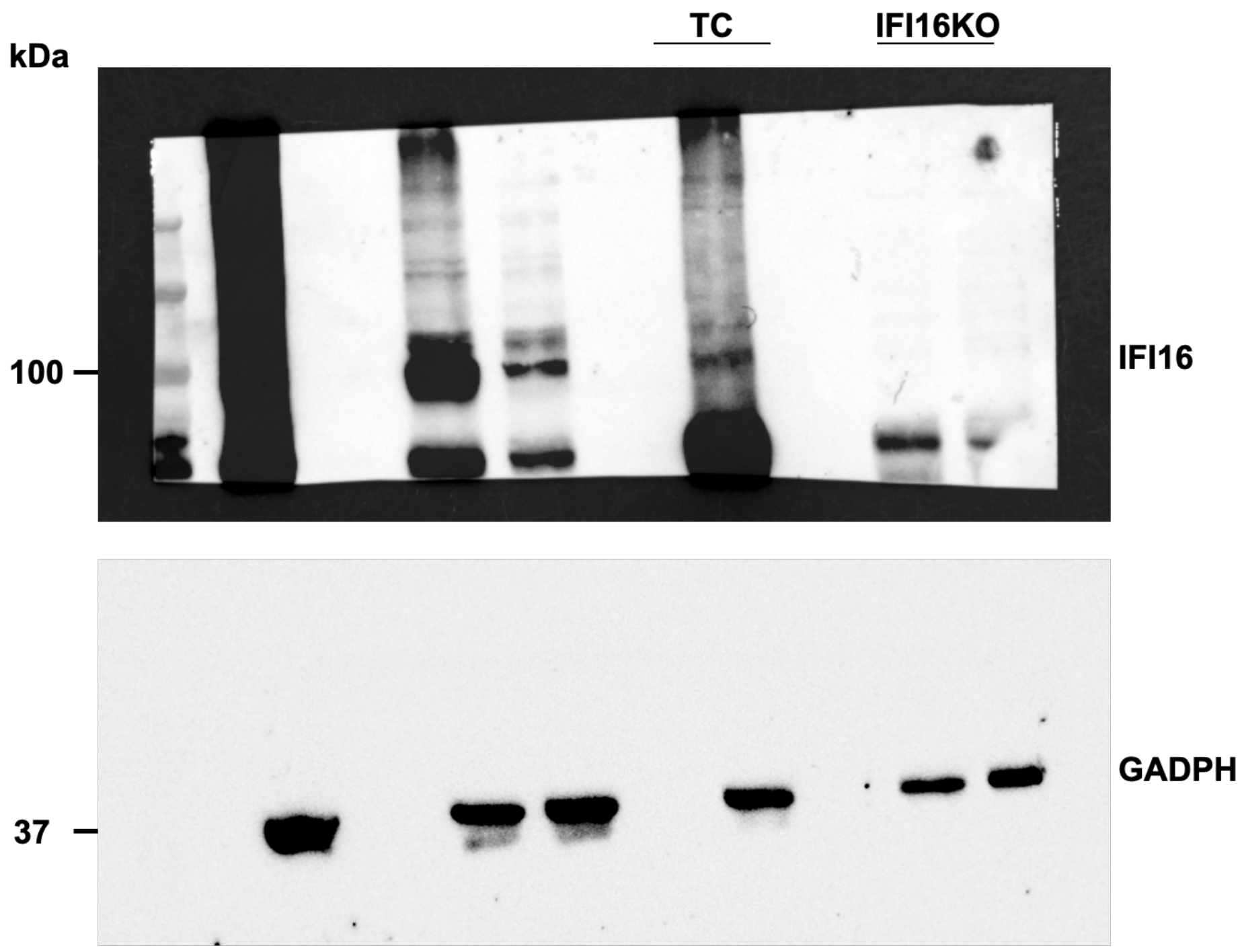

Supplement: Supplementary file 1 [file 42003_2026_10363_MOESM1_ESM.pdf]
