# Supplementary material for: IFI16 restricts SARS-CoV-2 replication by disrupting nucleocapsid-driven phase separation
Source: Commun Biol. 2026 Jun 1;9:1255. doi: 10.1038/s42003-026-10363-0 (PMC13614959; doi:10.1038/s42003-026-10363-0)
Supplement: Supplementary file 2 [file 42003_2026_10363_MOESM2_ESM.pdf]

Description of Additional Supplementary Files

File name: Supplementary Data 1-6

Description: The source data behind the graphs in the paper
