# Supplementary material for: IFI16 restricts SARS-CoV-2 replication by disrupting nucleocapsid-driven phase separation
Source: Commun Biol. 2026 Jun 1;9:1255. doi: 10.1038/s42003-026-10363-0 (PMC13614959; doi:10.1038/s42003-026-10363-0)
Supplement: Supplementary file 3 [file 42003_2026_10363_MOESM4_ESM.pdf]

nature portfolio

Corresponding author(s): Cinzia Borgogna, Marisa Gariglio

Last updated by author(s): 2026 03 06

Reporting Summary

Nature Portfolio wishes to improve the reproducibility of the work that we publish. This form provides structure for consistency and transparency in reporting. For further information on Nature Portfolio policies, see our Editorial Policies and the Editorial Policy Checklist.

Statistics

For all statistical analyses, confirm that the following items are present in the figure legend, table legend, main text, or Methods section.

n/a	Confirmed	
☐	☒	The exact sample size (n) for each experimental group/condition, given as a discrete number and unit of measurement
☐	☒	A statement on whether measurements were taken from distinct samples or whether the same sample was measured repeatedly
☐	☒	The statistical test(s) used AND whether they are one- or two-sided<br>*Only common tests should be described solely by name; describe more complex techniques in the Methods section.*
☒	☐	A description of all covariates tested
☐	☒	A description of any assumptions or corrections, such as tests of normality and adjustment for multiple comparisons
☐	☒	A full description of the statistical parameters including central tendency (e.g. means) or other basic estimates (e.g. regression coefficient) AND variation (e.g. standard deviation) or associated estimates of uncertainty (e.g. confidence intervals)
☐	☒	For null hypothesis testing, the test statistic (e.g. F, t, r) with confidence intervals, effect sizes, degrees of freedom and P value noted<br>*Give P values as exact values whenever suitable.*
☒	☐	For Bayesian analysis, information on the choice of priors and Markov chain Monte Carlo settings
☒	☐	For hierarchical and complex designs, identification of the appropriate level for tests and full reporting of outcomes
☐	☒	Estimates of effect sizes (e.g. Cohen's d, Pearson's r), indicating how they were calculated

Our web collection on statistics for biologists contains articles on many of the points above.

Software and code

Policy information about availability of computer code

Data collection	CFX96 Real-Time PCR Detection System (Bio-Rad Laboratories), Bio-Rad QX200 (Bio-Rad Laboratories), ChemiDoc Touch Imaging System (Bio-Rad Laboratories), Zeiss LSM 900 with Airyscan detector (Carl Zeiss), equipped with GaAsP detectors (Gallium:Arsenide:Phosphide), AxioScan 7 Microscope Slide Scanner (Carl Zeiss), Biacore X100 (GE Healthcare), NanoDrop spectrophotometer (ThermoFisher Scientific), Leica SP8 lightning confocal microscope (Leica Microsystems), Leica DM6000 B microscope (Leica Microsystems).
Data analysis	Fiji (NIH, Bethesda), Graph-Pad Prism version 7.00 for Windows (GraphPad Software), Real-time data were evaluated with CFX Maestro Software (Bio-Rad Laboratories), QuantaSoft software (Bio-Rad), LAS X software (Leica Microsystems).

For manuscripts utilizing custom algorithms or software that are central to the research but not yet described in published literature, software must be made available to editors and reviewers. We strongly encourage code deposition in a community repository (e.g. GitHub). See the Nature Portfolio guidelines for submitting code & software for further information.

Data

Policy information about availability of data

All manuscripts must include a data availability statement. This statement should provide the following information, where applicable:

- Accession codes, unique identifiers, or web links for publicly available datasets
- A description of any restrictions on data availability
- For clinical datasets or third party data, please ensure that the statement adheres to our policy

Numerical data from the analyses are available in the Supplementary Materials (Supplementary Data). All other data are available from the corresponding author upon reasonable request.

Research involving human participants, their data, or biological material

Policy information about studies with human participants or human data. See also policy information about sex, gender (identity/presentation), and sexual orientation and race, ethnicity and racism.

Reporting on sex and gender	NA
Reporting on race, ethnicity, or other socially relevant groupings	NA
Population characteristics	NA
Recruitment	NA
Ethics oversight	NA

Note that full information on the approval of the study protocol must also be provided in the manuscript.

Field-specific reporting

Please select the one below that is the best fit for your research. If you are not sure, read the appropriate sections before making your selection.

☒ Life sciences ☐ Behavioural & social sciences ☐ Ecological, evolutionary & environmental sciences

For a reference copy of the document with all sections, see nature.com/documents/nr-reporting-summary-flat.pdf

Life sciences study design

All studies must disclose on these points even when the disclosure is negative.

Sample size	Sample size was determined based on experimental feasibility and the need to obtain sufficient statistical power. Unless otherwise indicated, experiments were performed in three independent biological replicates (n = 3).
Data exclusions	Data were generally not excluded from the analyses
Replication	All data presented were obtained from three independent biological replicates, as indicated in the figure legends.
Randomization	Randomization was not used.
Blinding	Blinding was not applied because the experiments were performed using defined in vitro experimental conditions and quantitative molecular assays. However, all experiments and analysis were performed objective and unbiased.

Reporting for specific materials, systems and methods

We require information from authors about some types of materials, experimental systems and methods used in many studies. Here, indicate whether each material, system or method listed is relevant to your study. If you are not sure if a list item applies to your research, read the appropriate section before selecting a response.

Materials & experimental systems

n/a	Involved in the study
☐	☒ Antibodies
☐	☒ Eukaryotic cell lines
☒	☐ Palaeontology and archaeology
☒	☐ Animals and other organisms
☒	☐ Clinical data
☒	☐ Dual use research of concern
☒	☐ Plants

Methods

n/a	Involved in the study
☒	☐ ChIP-seq
☒	☐ Flow cytometry
☒	☐ MRI-based neuroimaging

Antibodies

Antibodies used

mouse mAbs anti-IFI16 (sc-8023; Santa Cruz), anti-dsRNA (MABE1134; Sigma-Aldrich), anti-SARS-CoV-2 S (GTX632604; GeneTex), rabbit mAb anti-SARS-CoV-2 N (40143-R001; SinoBiological), goat anti-mouse IgG-Alexa Fluor 488 (A11001; Thermo Fisher Scientific), goat anti-mouse IgG-Alexa Fluor 568 (A11004; Thermo Fisher Scientific), goat anti-rabbit IgG-Alexa Fluor 488 (A11008; Thermo Fisher Scientific), goat anti-rabbit IgG-Alexa Fluor 568 (A11011; Thermo Fisher Scientific), anti-IgG (C15410206; Diagenode), rabbit polyclonal antibodies (pAbs) anti-IFI16 (in-house made) and anti-GAPDH (60004-1-Ig; Proteintech).

Validation

Validation was provided by the vendor for all antibodies used in this study. In house-produced rabbit polyclonal anti-IFI16 was validated by previous users (doi: 10.1089/107999002320271413)

Eukaryotic cell lines

Policy information about cell lines and Sex and Gender in Research

Cell line source(s)

Rhesus monkey kidney epithelial cells (LLC-MK2, ATCC: CCL-7), African green monkey cells (Vero E6, ATCC: CRL-1586), human colorectal cancer cells (Caco-2,ATCC: HTB-37), human lung fibroblasts (MRC-5, IRCCS Ospedale Policlinico San Martino) and human lung adenocarcinoma cells (Calu-3, ATCC: HTB-55)

Authentication

Cell lines were obtained from commercial sources and used according to the supplier's specifications.

Mycoplasma contamination

We confirm that all cell lines tested negative for mycoplasma contamination.

Commonly misidentified lines
(See ICLAC register)

No commonly misidentified cell lines were used in the study.

Plants

Seed stocks

NA

Novel plant genotypes

NA

Authentication

NA
