# Supplementary material for: IFI16 restricts SARS-CoV-2 replication by disrupting nucleocapsid-driven phase separation
Source: Commun Biol. 2026 Jun 1;9:1255. doi: 10.1038/s42003-026-10363-0 (PMC13614959; doi:10.1038/s42003-026-10363-0)
Supplement: Supplementary file 4 [file 42003_2026_10363_MOESM5_ESM.pdf]

nature portfolio

Peer Review File

IFI16 restricts SARS CoV 2 replication by disrupting nucleocapsid-driven phase separation

Corresponding Author: Professor Cinzia Borgogna

This file contains all reviewer reports in order by version, followed by all author rebuttals in order by version.

Version 0:

Reviewer comments:

Reviewer #1

(Remarks to the Author)
The current manuscript introduces an interferon independent antiviral function of IFI16 against SARS-CoV-2 replication by antagonizing liquid-liquid phase separation of N proteins, which is required for proper viral replication. The investigation covers experiments with viral infection and in vitro analysis. IFI16 is a SARS-CoV-2- and OC43- specific restriction factor. In the case of NL63, IFI16 was found to be a proviral factor. The mechanism underlying its proviral function to NL63 was not clearly defined. Only one cell line (LLC-MK2) was tested. Moreover, it was postulated that the sequence of N-IDR of N proteins defines the subtype-dependent function of IFI16 in beta-coronavirus (IFI16 is antiviral) and alpha-coronavirus (IFI16 is proviral) (Figure 5). The postulation was only confirmed by testing the effect of IFI16 in few naturally occurring subtypes SARS-CoV-2 and OC43 vs NL63. Other strains were not tested. No recombinant virus experiment was performed to confirm the role of N-IDR. How IFI16 switches roles between coronavirus subtypes is only hypothetical. Some concerns should be solved before it is acceptable for the central idea of the manuscript.

1. It is misleading to say IFI16 differentially modulates coronavirus replication by disrupting N-driven LLPS in the title. No data shows that IFI16 promotes N-driven LLPS for NL63. It was only shown that IFI16 may disintegrate SARS-CoV-2 N-driven LLPS.

2. IFI16 is an interferon stimulated gene of some cell types. LLC-MK2 cells do not respond for early production of IFN upon SARS-CoV-2 and NL63 infection but express endogenous level of IFI16 which was not increased upon infection (Figure 2g, 2h vs 1c and 1d). Whether IFN induces the antiviral or proviral functions was not confirmed. In the abstract section, it is too early to say IFI16 interferes with coronavirus replication in an interferon-independent manner unless more general evidence shows IFN cannot pronounce the observed functions of IFI16 in other cell types and conditions. IFI16 may function independently to IFN in LLC-MK2. Whether the observed functions of IFI16 can be pronounced by extrinsic IFN, modulated by IFNAR1 knockout or observed in another cell lines proficient with IFN response should be tested to claim interferon-independent functions of IFI16.

3. The whole investigation relies heavily on IFI16 KO of LLC-MK2 cells. It will be important to validate the antiviral and proviral functions of IFI16 in human cells for the human coronaviruses.

4. NL63 was only tested for increased RNA replication and viral protein expression in IFI16 proficient LLC-MK2 cells (Figure 1 and 2). A difficulty was expressed for detecting NL63's infectious titre with CPE-based assay such as plaque assay or TCID-50 (line 138-139). It is suggested to perform immunostaining-based assays such as FFA to confirm if IFI16 promotes overall replication of NL63. The difference observed for RNA detection and protein detection was low but observable (Figure 1b, 1d, 1f, 2b and 2d).

5. Were all experiments validated by the two knockout clones shown in supplementary Figure 2? It will be important to confirm the functions of IFI16 by a second clone to reject potential clonal effects. It will be great to reconstitute expression of IFI16 in the knockout clones to test if the deprived function of IFI16 can be reverted.

6. In vitro experiment confirms the functions of IFI16 protein in disintegrating N from LLPS (Figure 4b-4d). Only IF staining was performed to confirm its role in infected cells (Figure 4e). The observed perinuclear structure of N in IFI16 KO cells was, however, not identified clearly to represent LLPS. E.g. whether the perinuclear structures of N proteins colocalize better with

G3BP1 which is known to interact with N in stress granule (PMID: 39638802 and 33479219).

7. Technically, it is important to compare protein detection level on the same immunoblot to prevent variation of blot exposure. It is difficult to judge if the repressed N and/or S observed in Figure 1c and 1b was due to membrane exposure or actual difference in protein level. Relative densitometry quantification may help.

8. It will be great to label Figure 1a-1d for whether SARS-CoV-2 or NL63 was tested. For Figure 2g-2h, the scale of Y-axis should be adjusted to illustrate most data points.

Reviewer #2

(Remarks to the Author)
In this manuscript Cislaghi et al., describe a disparate role for the interferon-inducible protein IFI16 during SARS-CoV-2 and NL63 infection of LLC-MK2 monkey epithelial cells. They find that SARS-CoV-2 replication is enhanced in IFI16 KO cells, whereas NL63 replication is decreased. The authors show that IFI16 forms a complex with the SARS-CoV-2 N protein in vitro and that IFI16, N, and viral genomic RNA interact during SARS-CoV-2 infection. They also show that IFI16 disrupts the LLPS of SARS-CoV-2 N both in vitro and speculate, based on super resolution microscopy, that it does so in vivo. The authors hypothesize that, because NL63 N lacks the N-terminal IDR that drives N protein condensation, this explains why IFI16 is not antiviral during NL63 infection.

Overall, the data is well presented, the manuscript is written clearly, and the workflow is easy to follow. This work is the first to extensively characterize of the role of IFI16 during human coronavirus infection and therefore of interest to the field, however several conclusions must be strengthened prior to publication.

Major Comments:

The authors must complement the KO cells by supplying IFI16 back prior to infection with both SARS-CoV-2 and NL63 and look for a reversal of their phenotype. The authors have previously shown they are capable of transducing cells to overexpress IFI16 (Cigno et al., JVI, 2015). This experiment is critical to support that no off-target mutations occurred during CRISPR/cas9 gene editing (Pacesa et al., Cell, 2023) or were accidentally selected for during single cell expansion.

The use of monkey epithelial cells to compare between SARS-CoV-2 and NL63 infection is somewhat justified. However, since the vast majority of the manuscript focuses on the mechanism of IFI16 antiviral activity during SARS-CoV-2 infection, these results should be repeated in a relevant human cell line. The generation of a KO-cell line is time consuming but, as the authors report a robust phenotype, it should be easily reproducible using siRNA-mediated IFI16 targeting as they also have done previously (Cigno et al., JVI, 2015).

The claim that this mechanism is independent of IFN production is poorly supported. The authors show that there is a statistically significant decrease in IFN production during SARS-CoV-2 infection of IFI16 KO cells. The justification that the same occurs during NL63 infection is poor rationale for excluding this hypothesis entirely. This can be easily addressed by using a JAK/STAT inhibitor or actinomycin D to inhibit cellular transcription as in previous work on IFI16 (Kim et al., Mol Cell, 2021).

The claim that the proviral and antiviral activities of IFI16 can be explained solely due to the presence or absence of an N-terminal IDR is not well supported experimentally. The is especially important since the authors write that lack of this IDR shifts IFI16 function from proviral to antiviral in the abstract of the manuscript. To strengthen this claim the authors should show that either a) that NL63 N does not interact with IFI16 or b) that deletion of the SARS-CoV-2 N-terminal IDR abolishes this interaction.

Minor comments:

Figure 1 - direct comparisons between N protein accumulation should be run on the same blot
Figure 3e - there are noticeable changes in the N protein banding pattern between the different conditions of the IP, could the authors please speculate as to why this is?
Figure 3g - given that the viral genome is not present in mock cells, normalizing to mock does not seem appropriate here
Figure 4e-h - the observed differences in N protein condensation could easily be explained by increased N protein expression in IFI16 KO cells (Figure 1), therefore this data is not convincing
Supplemental Figure 1a - these graphs should have labels to easily distinguish which corresponds to SARS-CoV-2 and which corresponds to NL63
Line 81-87 - this is speculative and belongs in the discussion not the introduction
Line 97-99 - these results should be described
Line 191 - please explain how your primers distinguish between genomic and subgenomic RNA

Reviewer #3

(Remarks to the Author)
The manuscript by Cislaghi et al. examines the interaction of the interferon gamma-inducible protein 16 (IFI16) during

coronavirus infection. Based on the observation that the SARS-CoV-2 N protein operates through liquid-liquid phase separation (LLPS) and IFI16 displays an IDR that can regulate LLPS they generated knockout cells for IFI16 and used these to investigate the potential role in virus replication of SARS-CoV-2 and NL63. They show that SARS-CoV-2 replication is increased on IFI16 KO cells while NL63 seems reduced. They continue to focus on SARS-CoV-2 and show that cytoplasmic IFI16 interacts directly with the N protein and vRNA resulting in interaction of IFI16 in the LLPS condensates in an IFN-independent manner. This interaction if removed by IFI16 knockout seems to increase SARS-CoV-2 replication. An interesting observation with mechanistically interrogation that discovers additional roles of a classical DNA sensor. While interesting for the readers, there are a few flaws that require further investigation to firmly come to the conclusions drawn by the authors.

Major:
- All work was carried out on LLC-MK2 cells. Not only is this a rhesus macaque cell line but also isolated from the kidney so not a primary target cell of coronavirus infection. The observation of IFI16 interaction with SARS-CoV-2 should be confirmed on human cells to show this is a broader observation and not localized to LLC-MK2 cells.
- I do not think a proviral role of IFI16 during NL63 replication can be concluded from the current results. Especially without infectious virus results.
- The inclusion of NL63 is not completely clear to this reviewer. Would it be better to focus on SARS-CoV-2 and show in the end that NL63 and OC43 behave differently?
- The effect of IFI16 on infectious virus needs stronger evidence. Figure 2 e-f shows plaque assay results but the plaques in Fig2e are not visible and the quantification in Fig2f shows a minor 0.5log change between KO and control cells. The biological relevance of this difference (even if significant) is questionable.
- The connection of IFI16 loss in bats needs better explanation. Based on the shown results the loss of IFI16 in bats would result in increased viral replication of SARS-CoV-2 in the bat reservoir. How would this connect to asymptomatic animals if there is more replication of the virus? Have you tested bat cells to show similar results as IFI16 KO cells?
- Supplement: Please show whole blots in the supplement.

Minor:
- Line 83: NL63 is not circulating in bats, this is a human virus. Please be clearer if you mean these viruses have an origin in bats.
- Line 101: The control cells are labelled as “transfected control” but as I understand it the KO cells were produced using lentiviral preparations. Would transduced control or non-targeting control be a better description?
- Line 115: Please specify abbreviation “pi”
- Line 117-18: Can you explain why you observe a drop in NL replication at 72 hours compared to 24 hours?
- Supplement Figure 1: Why are two different MOIs being used for SARS-CoV-2 and NL63 infections?
- Line 190 and Fig3f: Why is there SARS-CoV-2 N protein visible in the uninfected control IP for IFI16? And if its visible on the blot, why is this not shown in the quantification graph of 3g?
- Line 193 and Fig. 3g: fold change measured to what?
- Line 248: I do not think we can conclude that IFI16 supports NL63 replication.
- Line 278: This manuscript does not contain any “in vivo” approaches.
- Line 318: If IFI16 favors formation of membraneless organelles, would you expect that viral replication is more sensitive to other viral sensors within the cell?

Version 1:

Reviewer comments:

Reviewer #1

(Remarks to the Author)
The study is much improved by incorporating the use of IFI16 knock-in and a human cell line Calu-3 in addition to a new arrangement of the argument. The following are some new comments and suggestions:
1. How to explain IFI16 KO promotes early SARS-2 S but not N expression in LLC-MK2 cells (Figure 1c)? Quantification of the relative protein bands of independent experiments will enhance the study.
2. It would be great to measure and describe the colocalization of IFI16/N in the context of Calu-3 cells to support the findings in LLC-MK2 cells. It seems that IFI16/N do colocalize in Calu-3 cells as shown by figure 3d. The interpretation will facilitate and support the argument of the paper.
3. The viral RNA independent binding of IFI16 to N protein is uncertain (Figure 4e vs line 188-189). It would be great to provide quantification of the immunoblot of independent experiments showing RNase treatment does not affect the enrichment of N in the IFI16 immunoprecipitate. Alternatively, authors may consider to check if IFI16 binds with N when N is expressed by plasmids (i.e. without virus). In vitro pull-down experiment can also be considered.
4. Do you expect IFI16 binding to vRNA depend on N protein? Imaging to IFI16/dsRNA may be helpful to address the question, at least in part. Does IFI16 select specific viral RNA species (e.g. if IFI16 suppresses spike gene expression but not N gene (Figure 1c), would it binds more to the sgRNA or RNAs encoding spike gene?)
5. Many figures rely on quantification of imaging (e.g. Figure 2b, 2c, 3e, 3f, 5f-h). To enhance the transparency of the study, it would be great to provide the original pictures as supporting materials.
Some minor comments:
6. Line 155, figure 2f should be corrected to 3f.
7. The label for figure 4f upper blot is missing (IFI16?)

8. Please clarify the fold change in the below sentence if IgG was not set as 1 in Figure 4g
“Interaction between IFI16 and viral RNA. All viral genes analyzed showed increased expression in the infected-cell pull-down, with a statistically significant 3-fold increase for the genomic ORF1ab 205 gene ($p = 0.0006$) and a 4-fold upregulation for the genomic N gene ($p < 0.0001$). (line 203-205)”
9. Figure 5d requires clarification. Why Ctrl would have IFI16 signal?

Reviewer #2

(Remarks to the Author)
The authors have sufficiently addressed my concerns.

Reviewer #3

(Remarks to the Author)
The revised manuscript by Cislaghi et al. shows improved clarity and structure. The conclusions are more nuanced and fit the underlying data. Inclusion of an additional human cell line and the IFI16 complementation into the KO cells is strongly valued. As a general comment, looking at infectious virus instead of genome copies is preferred. Particularly when talking about restriction of viruses. Nonetheless, the changes in genome copies are influenced by IFI16 which is supported by this data. Overall, the authors addressed the raised comments and questions from my side.

Open Access This Peer Review File is licensed under a Creative Commons Attribution 4.0 International License, which permits use, sharing, adaptation, distribution and reproduction in any medium or format, as long as you give appropriate credit to the original author(s) and the source, provide a link to the Creative Commons license, and indicate if changes were made.
In cases where reviewers are anonymous, credit should be given to 'Anonymous Referee' and the source.
The images or other third party material in this Peer Review File are included in the article’s Creative Commons license, unless indicated otherwise in a credit line to the material. If material is not included in the article’s Creative Commons license and your intended use is not permitted by statutory regulation or exceeds the permitted use, you will need to obtain permission directly from the copyright holder.
To view a copy of this license, visit https://creativecommons.org/licenses/by/4.0/

Reviewers' comments:

Reviewer #1 (Remarks to the Author):

The current manuscript introduces an interferon independent antiviral function of IFI16 against SARS-CoV-2 replication by antagonizing liquid-liquid phase separation of N proteins, which is required for proper viral replication. The investigation covers experiments with viral infection and in vitro analysis. IFI16 is a SARS-CoV-2- and OC43- specific restriction factor. In the case of NL63, IFI16 was found to be a proviral factor. The mechanism underlying its proviral function to NL63 was not clearly defined. Only one cell line (LLC-MK2) was tested. Moreover, it was postulated that the sequence of N-IDR of N proteins defines the subtype-dependent function of IFI16 in beta-coronavirus (IFI16 is antiviral) and alpha-coronavirus (IFI16 is proviral) (Figure 5). The postulation was only confirmed by testing the effect of IFI16 in few naturally occurring subtypes SARS-CoV-2 and OC43 vs NL63. Other strains were not tested. No recombinant virus experiment was performed to confirm the role of N-IDR. How IFI16 switches roles between coronavirus subtypes is only hypothetical. Some concerns should be solved before it is acceptable for the central idea of the manuscript.

1. It is misleading to say IFI16 differentially modulates coronavirus replication by disrupting N-driven LLPS in the title. No data shows that IFI16 promotes N-driven LLPS for NL63. It was only shown that IFI16 may disintegrate SARS-CoV-2 N-driven LLPS.

We thank the reviewer for this valuable comment. The title has been revised to read: “The interferon-inducible IFI16 restricts SARS-CoV-2 replication via disruption of nucleocapsid-driven liquid–liquid phase separation.”

2. IFI16 is an interferon stimulated gene of some cell types. LLC-MK2 cells do not respond for early production of IFN upon SARS-CoV-2 and NL63 infection but express endogenous level of IFI16 which was not increased upon infection (Figure 2g, 2h vs 1c and 1d). Whether IFN induces the antiviral or proviral functions was not confirmed. In the abstract section, it is too early to say IFI16 interferes with coronavirus replication in an interferon-independent manner unless more general evidence shows IFN cannot pronounce the observed functions of IFI16 in other cell types and conditions. IFI16 may function independently to IFN in LLC-MK2. Whether the observed functions of IFI16 can be pronounced by extrinsic IFN, modulated by IFNAR1 knockout or observed in another cell lines proficient with IFN response should be tested to claim interferon-independent functions of IFI16.

We appreciate this point. In our LLC-MK2 model, under the conditions used, we did not detect a significant induction of type I IFNs or interferon-stimulated genes upon SARS-CoV-2 infection in either TC or IFI16KO cells (Figure 2f), and IFI16 levels were not increased upon infection (Figure 1c). Therefore, our data do not support a role for endogenous IFN production in mediating the observed IFI16-dependent phenotype in this system. To avoid overinterpretation, we revised the Results and Discussion accordingly (page 6, lines 135–140; pages 12-13, lines 301–304) and removed any reference to “interferon-independent” activity from the Abstract.

3. The whole investigation relies heavily on IFI16 KO of LLC-MK2 cells. It will be important to validate the antiviral and proviral functions of IFI16 in human cells for the human coronaviruses.

We thank the reviewer for this important suggestion. We extended the analysis to human Calu-3 lung epithelial cells. IFI16 was depleted (siRNA) prior to SARS-CoV-2 infection, and viral replication was assessed at 24 and 48 hpi. IFI16 depletion increased the fraction of N-positive and dsRNA-

positive cells by immunofluorescence and increased viral genome copies in culture supernatants measured by ddPCR, consistent with our observations in LLC-MK2 cells. These new data have been added to the revised manuscript (page 7, lines 149–157; Figure 3d–g).

4. *NL63 was only tested for increased RNA replication and viral protein expression in IFI16 proficient LLC-MK2 cells (Figure 1 and 2). A difficulty was expressed for detecting NL63's infectious titre with CPE-based assay such as plaque assay or TCID-50 (line 138-139). It is suggested to perform immunostaining-based assays such as FFA to confirm if IFI16 promotes overall replication of NL63. The difference observed for RNA detection and protein detection was low but observable (Figure 1b, 1d, 1f, 2b and 2d).*

As suggested by Reviewer 3, we revised the manuscript to further focus the study on SARS-CoV-2. Accordingly, the NL63 results have been moved to a dedicated paragraph at the end of the Results. In this section, we assessed whether IFI16 also modulates the replication of low-pathogenicity coronaviruses by infecting IFI16KO and control cells with OC43 and NL63 and quantifying viral genome copies in culture supernatants by ddPCR (Figure 6a–b).

5. Were all experiments validated by the two knockout clones shown in supplementary Figure 2? It will be important to confirm the functions of IFI16 by a second clone to reject potential clonal effects. It will be great to reconstitute expression of IFI16 in the knockout clones to test if the deprived function of IFI16 can be reverted.

We thank the reviewer for this important point. To address the concern about potential clonal effects, we performed complementation experiments by re-expressing IFI16 in IFI16KO cells using an IFI16–mCherry construct prior to SARS-CoV-2 infection. As described in the revised manuscript (page 6, lines 141–148; Figure 3a–c), IFI16 re-expression reduced dsRNA foci at 48 h post-infection (Fig. 3a–b) and was associated with decreased viral RNA levels in culture supernatants measured by ddPCR (Fig. 3c). These data provide independent support that the phenotype observed in IFI16KO cells is IFI16-dependent.

6. In vitro experiment confirms the functions of IFI16 protein in disintegrating N from LLPS (Figure 4b-4d). Only IF staining was performed to confirm its role in infected cells (Figure 4e). The observed perinuclear structure of N in IFI16 KO cells was, however, not identified clearly to represent LLPS. E.g. whether the perinuclear structures of N proteins colocalize better with G3BP1 which is known to interact with N in stress granule (PMID: 39638802 and 33479219).

We thank the reviewer for this helpful suggestion. To assess whether the perinuclear N-positive structures observed in IFI16KO cells correspond to stress granules, we examined the localization of the stress granule marker G3BP1. Under our experimental conditions and at all analyzed time points, G3BP1 did not accumulate in structures resembling stress granules and did not colocalize with the N-positive condensates (Figure below). Therefore, our data do not support the interpretation that these structures represent canonical stress granules. We have clarified this point and revised the corresponding text in the Discussion (page 13, lines 323–325). We now refer to these structures conservatively as N-positive condensates.

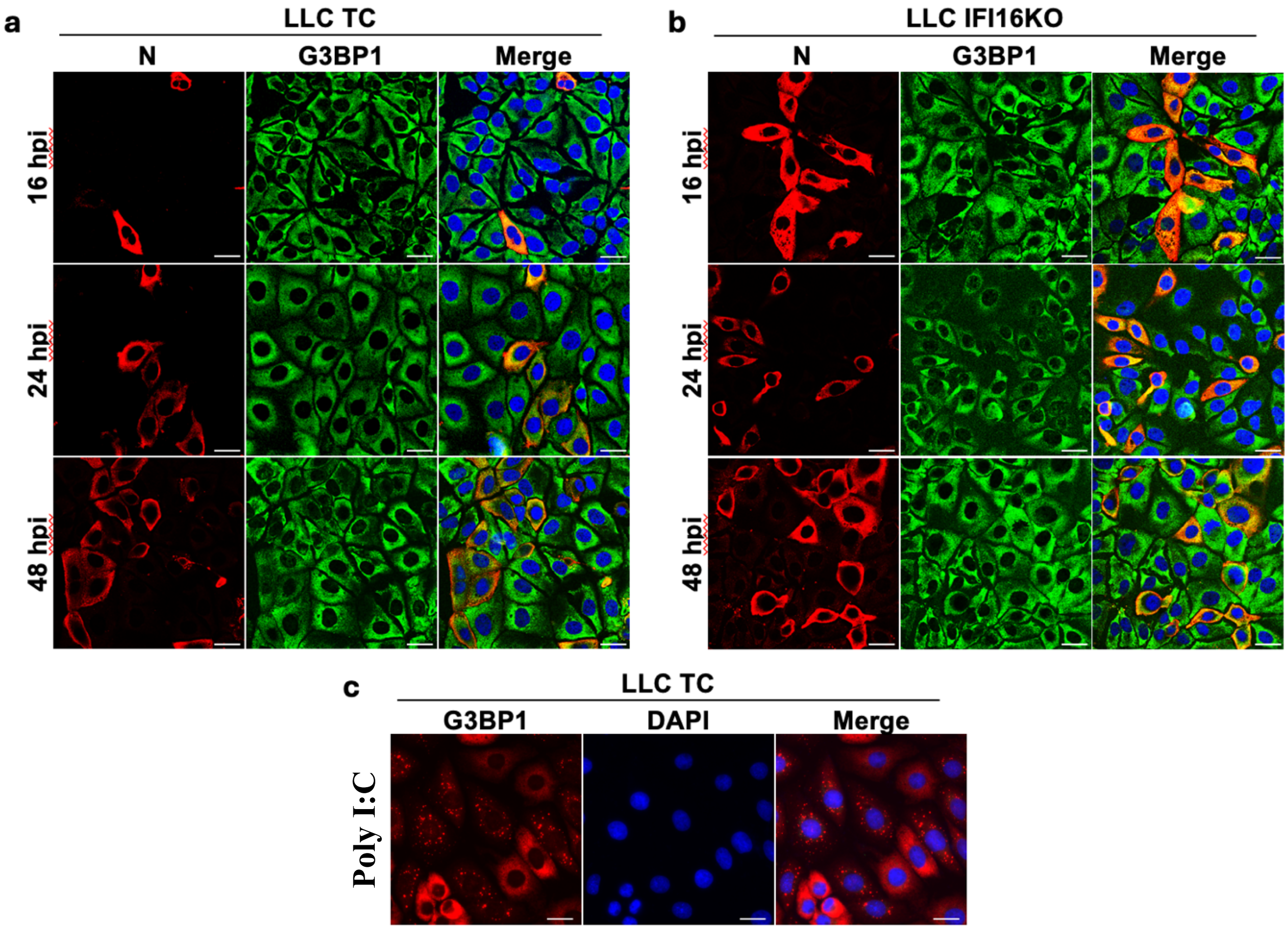

(a)TC and **(b)** IFI16KO cells were infected with SARS-CoV-2 at an MOI of 0.5. At the indicated hpi cells were processed for immunofluorescence analysis for N (red) and G3BP1 (green). Nuclei were visualized by DAPI (blue). **(c)** TC cells stimulated 7h with Poly I:C (Positive control). Cells were processed for immunofluorescence analysis for G3BP1 (red). Nuclei were visualized by DAPI (blue). Images were captured by confocal microscopy. Scale bars: 25 µm.

7. *Technically, it is important to compare protein detection level on the same immunoblot to prevent variation of blot exposure. It is difficult to judge if the repressed N and/or S observed in Figure 1c and 1b was due to membrane exposure or actual difference in protein level. Relative densitometry quantification may help.*

We thank the reviewer for this helpful suggestion. To ensure accurate comparison of protein levels, we repeated the immunoblot experiments loading lysates from LLC-MK2 TC and IFI16KO cells side by side on the same membrane for each time point. Samples were arranged so that direct comparisons were performed within the same blot (8 and 16 hpi in one blot; 24 and 48 hpi in another).

8. *It will be great to label Figure 1a-1d for whether SARS-CoV-2 or NL63 was tested. For Figure 2g-2h, the scale of Y-axis should be adjusted to illustrate most data points.*

We thank the reviewer for this suggestion. In the revised manuscript, Figure 1 has been reorganized and now exclusively presents SARS-CoV-2 data, with virus identity clearly indicated in the figure panels. In addition, as recommended, the Y-axis scale in Figure 2g (now Figure 2f) has been adjusted to better display the distribution of data points and improve visualization.

Reviewer #2 (Remarks to the Author):

In this manuscript Cislaghi et al., describe a disparate role for the interferon-inducible protein IFI16 during SARS-CoV-2 and NL63 infection of LLC-MK2 monkey epithelial cells. They find that SARS-CoV-2 replication is enhanced in IFI16 KO cells, whereas NL63 replication is decreased. The authors show that IFI16 forms a complex with the SARS-CoV-2 N protein in vitro and that IFI16, N, and viral genomic RNA interact during SARS-CoV-2 infection. They also show that IFI16 disrupts the LLPS of SARS-CoV-2 N both in vitro and speculate, based on super resolution microscopy, that it does so in vivo. The authors hypothesize that, because NL63 N lacks the N-terminal IDR that drives N protein condensation, this explains why IFI16 is not antiviral during NL63 infection.

Overall, the data is well presented, the manuscript is written clearly, and the workflow is easy to follow. This work is the first to extensively characterize of the role of IFI16 during human coronavirus infection and therefore of interest to the field, however several conclusions must be strengthened prior to publication.

Major Comments:

1. The authors must complement the KO cells by supplying IFI16 back prior to infection with both SARS-CoV-2 and NL63 and look for a reversal of their phenotype. The authors have previously shown they are capable of transducing cells to overexpress IFI16 (Cigno et al., JVI, 2015). This experiment is critical to support that no off-target mutations occurred during CRISPR/cas9 gene editing (Pacesa et al., Cell, 2023) or were accidentally selected for during single cell expansion.

We thank the reviewer for this important comment. To address this point, we performed complementation experiments by re-expressing IFI16 in IFI16KO cells prior to infection. Re-expression reduced dsRNA foci and viral RNA levels, supporting that the phenotype observed in IFI16KO cells is IFI16-dependent. These data are now included in the revised manuscript (page 6, lines 141–148; Figure 3a–c).

2. The use of monkey epithelial cells to compare between SARS-CoV-2 and NL63 infection is somewhat justified. However, since the vast majority of the manuscript focuses on the mechanism of IFI16 antiviral activity during SARS-CoV-2 infection, these results should be repeated in a relevant human cell line. The generation of a KO-cell line is time consuming but, as the authors report a robust phenotype, it should be easily reproducible using siRNA-mediated IFI16 targeting as they also have done previously (Cigno et al., JVI, 2015).

We thank the reviewer for this suggestion. The relevance of our findings is further supported by extending the analysis to human Calu-3 lung epithelial cells. IFI16 depletion prior to SARS-CoV-2 infection increased the fraction of infected cells and viral genome copies, consistent with results obtained in LLC-MK2 cells. These data have been added to the revised manuscript (pages 6-7, lines 149–157; Figure 3d–g).

3. The claim that this mechanism is independent of IFN production is poorly supported. The authors show that there is a statistically significant decrease in IFN production during SARS-CoV-2 infection of IFI16 KO cells. The justification that the same occurs during NL63 infection is poor rationale for excluding this hypothesis entirely. This can be easily addressed by using a JAK/STAT inhibitor or actinomycin D to inhibit cellular transcription as in previous work on IFI16 (Kim et al., Mol Cell, 2021).

We appreciate this point. In our LLC-MK2 system, we did not detect significant induction of type I IFNs or ISGs upon infection, and IFI16 levels were not increased. Therefore, our data do not support

a role for endogenous IFN production in mediating the observed phenotype under these conditions. To avoid overinterpretation, we revised the Results and Discussion (page 6, lines 130–134; pages 12, lines 301–304) and removed statements referring to interferon-independent activity from the Abstract.

4. The claim that the proviral and antiviral activities of IFI16 can be explained solely due to the presence or absence of an N-terminal IDR is not well supported experimentally. The is especially important since the authors write that lack of this IDR shifts IFI16 function from proviral to antiviral in the abstract of the manuscript. To strengthen this claim the authors should show that either a) that NL63 N does not interact with IFI16) or b) that deletion of the SARS-CoV-2 N-terminal IDR abolishes this interaction.

We agree with the reviewer that our data do not experimentally demonstrate a causal role for the N-terminal intrinsically disordered region (IDR). Accordingly, we removed this interpretation from the Abstract and revised both the Results and Discussion sections (pages 12, lines 278-282; page 14, lines 348-351). We now state that our observations are correlative and that further experiments will be required to directly test the contribution of N-protein architecture.

Minor comments:

1. Figure 1 - direct comparisons between N protein accumulation should be run on the same blot

We thank the reviewer for this suggestion. We repeated the immunoblots loading TC and IFI16KO lysates side by side on the same membrane for each time point (Fig. 1c).

2. Figure 3e - there are noticeable changes in the N protein banding pattern between the different conditions of the IP, could the authors please speculate as to why this is?

We thank the reviewer for this observation. The differences in N banding likely reflect altered recovery of IFI16-containing complexes under different IP conditions. The manuscript text has been clarified accordingly (page 8, lines 188-191).

3. Figure 3g - given that the viral genome is not present in mock cells, normalizing to mock does not seem appropriate here

We thank the reviewer for pointing this out. The graph has been revised and values are now normalized to the IgG IP control within infected samples. The manuscript (page 9, line 202), figure (now Fig. 4g) and legend have been updated.

4. Figure 4e-h - the observed differences in N protein condensation could easily be explained by increased N protein expression in IFI16 KO cells (Figure 1), therefore this data is not convincing

We agree that increased N abundance may contribute to the phenotype. We did not quantify per-cell N levels and therefore cannot exclude this possibility. However, super-resolution imaging shows distinct spatial and temporal organization of N in IFI16KO versus control cells, including earlier formation of perinuclear condensates and increased colocalization with Spike protein. The text has been revised to clarify this interpretation.

5. Supplemental Figure 1a - these graphs should have labels to easily distinguish which corresponds to SARS-CoV-2 and which corresponds to NL63

The figures have been reorganized and virus identity is now clearly indicated in all panels. Relevant data have been moved to the main figures and described in the text.

6. Line 81-87 - this is speculative and belongs in the discussion not the introduction
We thank the reviewer for this observation. The speculative sentence has been removed from the Introduction.

7. Line 97-99 - these results should be described

These data have now been incorporated into the main text and are described in the Results section pages 4-5, lines 92-101, Fig. 1a-b).

8. Line 191 - please explain how your primers distinguish between genomic and subgenomic RNA

We have clarified in the Materials and Methods how primers distinguish genomic from subgenomic RNA (page 16, lines 401-402).

Reviewer #3 (Remarks to the Author):

The manuscript by Cislaghi et al. examines the interaction of the interferon gamma-inducible protein 16 (IFI16) during coronavirus infection. Based on the observation that the SARS-CoV-2 N protein operates through liquid-liquid phase separation (LLPS) and IFI16 displays an IDR that can regulate LLPS they generated knockout cells for IFI16 and used these to investigate the potential role in virus replication of SARS-CoV-2 and NL63. They show that SARS-CoV-2 replication is increased on IFI16 KO cells while NL63 seems reduced. They continue to focus on SARS-CoV-2 and show that cytoplasmic IFI16 interacts directly with the N protein and vRNA resulting in interaction of IFI16 in the LLPS condensates in an IFN-independent manner. This interaction if removed by IFI16 knockout seems to increase SARS-CoV-2 replication. An interesting observation with mechanistically interrogation that discovers additional roles of a classical DNA sensor. While interesting for the readers, there are a few flaws that require further investigation to firmly come to the conclusions drawn by the authors.

Major:

1. All work was carried out on LLC-MK2 cells. Not only is this a rhesus macaque cell line but also isolated from the kidney so not a primary target cell of coronavirus infection. The observation of IFI16 interaction with SARS-CoV-2 should be confirmed on human cells to show this is a broader observation and not localized to LLC-MK2 cells.

We thank the reviewer for this important suggestion. To address this point, we extended our analysis to human Calu-3 lung epithelial cells. IFI16 depletion increased infection levels and viral genome copies, consistent with observations obtained in LLC-MK2 cells. These data have been added to the revised manuscript (page 7, lines 149–157; Figure 3d–g).

2. I do not think a proviral role of IFI16 during NL63 replication can be concluded from the current results. Especially without infectious virus results.

3. The inclusion of NL63 is not completely clear to this reviewer. Would it be better to focus on SARS-CoV-2 and show in the end that NL63 and OC43 behave differently?

To address the concern raised in this comment and in the previous one (comment 2), we have revised the manuscript accordingly. The description of IFI16's effect on NL63 replication has been moved to a dedicated section towards the end of the Results, titled "IFI16 Differentially Modulates the Replication of the Low-Pathogenic Coronaviruses OC43 and NL63".

4. The effect of IFI16 on infectious virus needs stronger evidence. Figure 2 e-f shows plaque assay results but the plaques in Fig2e are not visible and the quantification in Fig2f shows a minor 0.5log change between KO and control cells. The biological relevance of this difference (even if significant) is questionable.

To improve data clarity, we have replaced the images in Figure 2e, in which the plaques are now clearly visible.
The plaque assay shows a statistically significant increase in infectious SARS-CoV-2 released from IFI16KO cells, and this result is confirmed by ddPCR (Figure 1d). Our aim was to quantify differences in the amount of virus produced and released by TC versus IFI16KO cells. The concordant increase detected in IFI16KO cells by two independent methods—plaque assay and ddPCR—supports the interpretation that IFI16 contributes to restricting SARS-CoV-2 replication under our experimental conditions.

5. The connection of IFI16 loss in bats needs better explanation. Based on the shown results the loss of IFI16 in bats would result in increased viral replication of SARS-CoV-2 in the bat reservoir. How would this connect to asymptomatic animals if there is more replication of the virus? Have you tested bat cells to show similar results as IFI16 KO cells?

We thank the reviewer for this insightful comment. As we have not performed experiments in bat-derived cell lines, and to avoid speculation not directly supported by our data, we have removed all comments related to IFI16 loss in bats from the revised manuscript.

6. Supplement: Please show whole blots in the supplement.

The uncropped, full Western blot images have now been included in the revised Supplementary Figure S5-6.

Minor:

1. Line 83: NL63 is not circulating in bats, this is a human virus. Please be clearer if you mean these viruses have an origin in bats.

We have removed the reference to bats from the main text. While HCoV-NL63 is a human coronavirus, phylogenetic analyses suggest a zoonotic origin from bat coronaviruses[1]. However, given the focus of this manuscript, we have opted to omit this evolutionary aspect to maintain clarity.

2. Line 101: The control cells are labelled as "transfected control" but as I understand it the KO cells were produced using lentiviral preparations. Would transduced control or non-targeting control be a better description?

We thank the reviewer for this important observation. Indeed, both the IFI16 knockout and control LLC-MK2 cell lines were generated using lentiviral transduction. To reflect this accurately, we have revised the terminology throughout the manuscript, replacing "transfected control" with "transduced control" LLC-MK2 cells.

3. Line 115: Please specify abbreviation "pi"

We have revised the manuscript to specify the abbreviation at its first occurrence, now reading *"post infection (pi)"* at page 5, line 113.

4. Line 117-18: Can you explain why you observe a drop in NL replication at 72 hours compared to 24 hours?

We thank the reviewer for this important observation. The apparent decrease in NL63 replication at 72 hpi compared to 24 hpi may be influenced by the presence of residual input virus at the earlier time point, as the culture medium was changed only at 24 hpi. This may have led to an overestimation of viral RNA levels at 24 hpi. To avoid this potential confounding factor and to better reflect productive viral replication, we have revised the manuscript to include only the 72 and 144 hpi time points, which more accurately represent newly produced virus.

5. Supplement Figure 1: Why are two different MOIs being used for SARS-CoV-2 and NL63 infections?

In our study, different MOIs were employed for each virus in order to achieve comparable number of infected cells in our cellular model. The MOIs used are clearly indicated in each figure legend throughout the manuscript and supplementary materials for clarity and reproducibility.

6. Line 190 and Fig3f: Why is there SARS-CoV-2 N protein visible in the uninfected control IP for IFI16? And if its visible on the blot, why is this not shown in the quantification graph of 3g?

The band visible in the uninfected control IP lane of Figure 3f (now Figure 4f) does not correspond to the SARS-CoV-2 N protein, but to the heavy chain of IgG (~50 kDa) derived from the antibody used in the immunoprecipitation. Importantly, no N protein is present in uninfected cells, as confirmed by the absence of SARS-CoV-2 RNA in the corresponding immunoprecipitates, as shown in Figure 4g. The quantification in Figure 4g refers specifically to SARS-CoV-2 RNA detected by qPCR, where uninfected controls consistently yielded no amplification.

7. Line 193 and Fig. 3g: fold change measured to what?

We thank the reviewer for pointing out this important clarification. The fold change shown in Figure 3g (now Figure 4g) was calculated relative to the isotype IgG control immunoprecipitation (IgG-IP) from the same infected-cell lysates. We have now amended the figure 4g and the figure legend to clarify this.

8. Line 248: I do not think we can conclude that IFI16 supports NL63 replication.

Our findings indicate a differential impact of IFI16 on the replication of different human coronaviruses. Specifically, while IFI16 deficiency enhances OC43 replication, it is associated with reduced NL63 replication in our experimental system. The molecular basis of this effect remains unclear, and additional studies will be required to determine how IFI16 influences NL63 replication in IFI16-proficient (TC) cells. We have revised the manuscript to reflect this interpretation more accurately (page 14, Lines 344-351).

9. Line 278: This manuscript does not contain any "in vivo" approaches.

In our original manuscript, the term "*in vivo*" was used to describe the cellular model versus the biochemical assays (such as SPR and turbidity measurements). However, to prevent misunderstanding, we have now removed the term "in vivo" from the manuscript. It has been replaced with "cellular model".

10. Line 318: If IFI16 favors formation of membraneless organelles, would you expect that viral replication is more sensitive to other viral sensors within the cell?

We appreciate this point. Our data indicate that loss of IFI16 is associated with increased formation of membraneless condensates that correlate with enhanced viral replication. However, our experiments specifically address IFI16, and additional studies would be required to determine whether other cellular sensors modulate viral replication under these conditions.

Bibliography

1. Huynh, J. *et al.* Evidence Supporting a Zoonotic Origin of Human Coronavirus Strain NL63. *J Virol* **86**, 12816–12825 (2012).

Reviewer #1 (Remarks to the Author):

The study is much improved by incorporating the use of IFI16 knock-in and a human cell line Calu-3 in addition to a new arrangement of the argument. The following are some new comments and suggestions:

1. How to explain IFI16 KO promotes early SARS-2 S but not N expression in LLC-MK2 cells (Figure 1c)? Quantification of the relative protein bands of independent experiments will enhance the study.

We thank the reviewer for this insightful comment. As discussed in the manuscript, the differential effect observed for S versus N protein expression likely reflects differences in their expression kinetics. Specifically, S protein becomes detectable earlier in IFI16KO cells, suggesting a shift in expression timing, whereas no comparable effect is observed for N protein. This pattern was consistently observed across independent biological replicates.
In response to the reviewer's suggestion, we performed band quantification of independent experiments. Although a modest increase in protein levels was observed in IFI16KO compared with control cells, these differences were limited and did not substantially add to the conclusions already supported by the qualitative analysis. For this reason, we have not included these data in the revised manuscript.

2. It would be great to measure and describe the colocalization of IFI16/N in the context of Calu-3 cells to support the findings in LLC-MK2 cells. It seems that IFI16/N do colocalize in Calu-3 cells as shown by figure 3d. The interpretation will facilitate and support the argument of the paper.

To address this point, we have now included higher-magnification images in Supplementary Figure 2 clearly showing the colocalization of IFI16 and the viral N protein in Calu-3 cells. This observation further supports the findings obtained in LLC-MK2 cells and has now been described in the revised manuscript (Page 8, Lines 175–177).

3. The viral RNA independent binding of IFI16 to N protein is uncertain (Figure 4e vs line 188-189). It would be great to provide quantification of the immunoblot of independent experiments showing RNase treatment does not affect the enrichment of N in the IFI16 immunoprecipitate. Alternatively, authors may consider to check if IFI16 binds with N when N is expressed by plasmids (i.e. without virus). In vitro pull-down experiment can also be considered.

To clarify this point, we would like to note that our conclusion is supported by the surface plasmon resonance (SPR) analysis shown in Figure 4d, which demonstrates a direct interaction between recombinant IFI16 and N protein in a cell-free system, i.e. in the absence of viral RNA. In our view, this provides strong evidence that IFI16 can bind N in an RNA-independent manner.

4. Do you expect IFI16 binding to vRNA depend on N protein? Imaging to IFI16/dsRNA may be helpful to address the question, at least in part.

We thank the reviewer for this thoughtful comment. At present, based on the data included in this manuscript, we cannot formally establish whether IFI16 binding to viral RNA depends

on the N protein. Notably, we have evidence from proximity ligation assay experiments in TC cells transfected with poly(I:C) for 8 h, used as a surrogate of dsRNA, supporting the ability of IFI16 to associate with dsRNA in the absence of viral infection. These observations suggest that IFI16 may interact with RNA independently of N. However, as these data are part of a separate study currently in preparation, we prefer not to include them in the present manuscript.

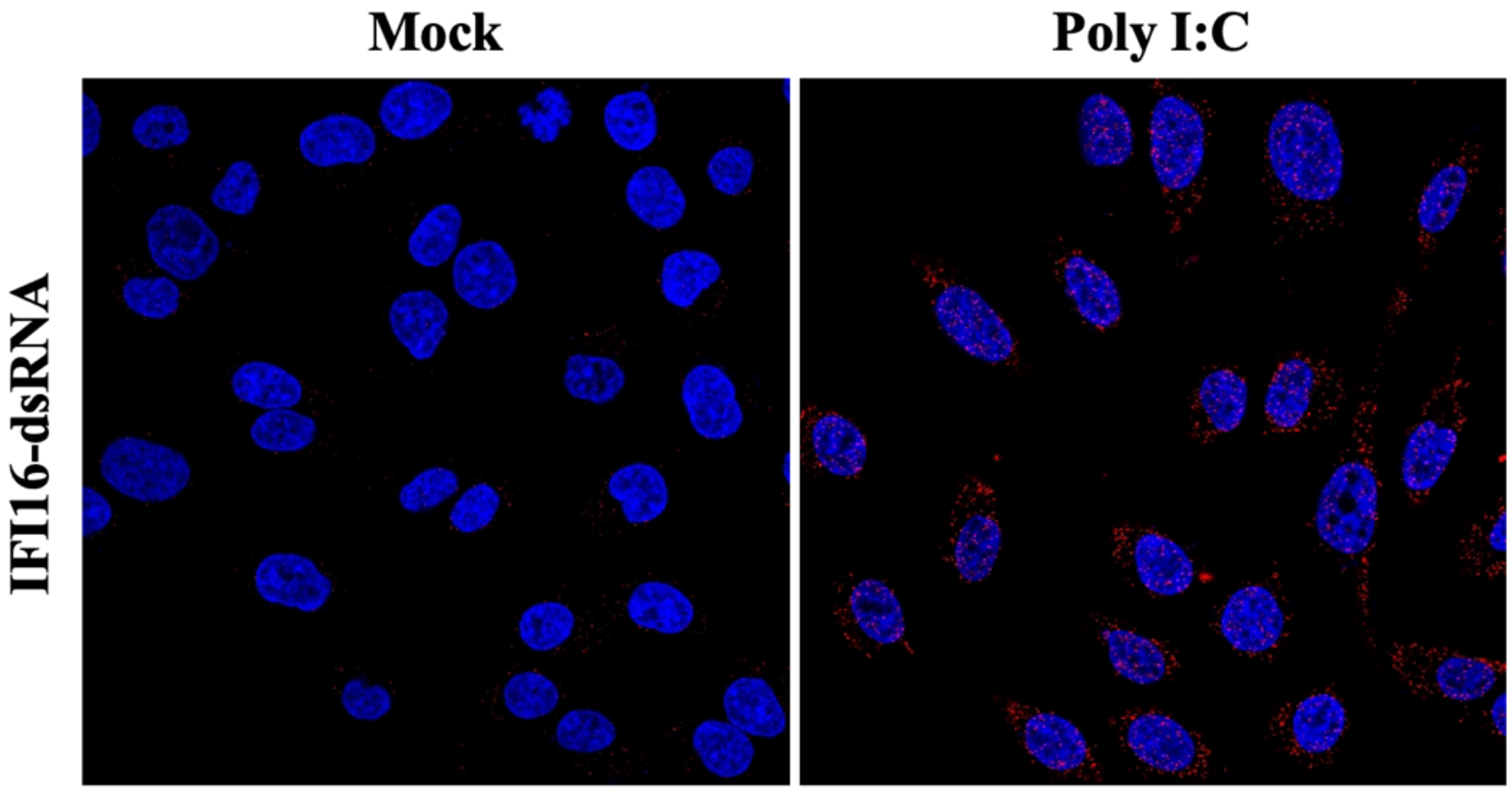

Figure 1. TC cells were transfected with poly(I:C) for 8 h and subsequently processed for PLA analysis using anti-IFI16 and anti-dsRNA antibodies. Nuclei were counterstained with DAPI (blue). Images were acquired using a Zeiss AxioScan.Z1 and are representative of three independent experiments.

Does IFI16 select specific viral RNA species (e.g. if IFI16 suppresses spike gene expression but not N gene (Figure 1c), would it binds more to the sgRNA or RNAs encoding spike gene?)

Current evidence indicates that IFI16 primarily recognizes nucleic acids in a largely sequence-independent manner, through its HIN domains, which bind DNA in a structure- and length-dependent rather than sequence-specific way (Unterholzner et al., 2010; Jin et al., 2012; Morrone et al., 2014). Although these studies mainly focus on DNA sensing, they support the notion that IFI16 binding is not strictly sequence-specific. In line with this, our data, including the observed binding to poly(I:C), are consistent with a similar mode of recognition for RNA. We agree that this is an interesting and relevant aspect; however, a detailed analysis of IFI16 binding to distinct viral RNA species is beyond the scope of the present study and will require dedicated approaches.

5. Many figures rely on quantification of imaging (e.g. Figure 2b, 2c, 3e, 3f, 5f-h). To enhance the transparency of the study, it would be great to provide the original pictures as supporting materials.

We have now specified in the manuscript that the original images will be made available upon request.

Some minor comments:

6. Line 155, figure 2f should be corrected to 3f.

The figure reference has now been corrected from Figure 2f to Figure 3f in the revised manuscript.

7. The label for figure 4f upper blot is missing (IFI16?)

The label in Figure 4f has now been added.

8. Please clarify the fold change in the below sentence if IgG was not set as 1 in Figure 4g "Interaction between IFI16 and viral RNA. All viral genes analyzed showed increased expression in the infected-cell pull-down, with a statistically significant 3-fold increase for the genomic ORF1ab gene (p = 0.0006) and a 4-fold upregulation for the genomic N gene (p < 0.0001). (line 203-205)"

To clarify this point, we have now added a statement in both the main text and the figure legend specifying that data are presented as log10-transformed fold enrichment relative to the IgG control (set to 0). We have also clarified that the reported 3-fold and 4-fold increases refer to values in linear scale.

9. Figure 5d requires clarification. Why Ctrl would have IFI16 signal?

In the control condition, the IFI16 signal arises from the fluorescently labeled IFI16 protein that is intentionally included in the assay at low concentration to monitor its localization. This labeled fraction allows visualization of IFI16 recruitment into phase-separated droplets without affecting droplet formation.
Importantly, the labeled protein is used at a very low concentration (0.15 µM), which does not influence droplet formation but enables detection of its distribution. This control condition is necessary to interpret the behavior of IFI16 in the experimental settings.

Bibliography

- Unterholzner, L. *et al.* IFI16 is an innate immune sensor for intracellular DNA. Nat. Immunol. 11, 997–1004 (2010).
- Jin, T. *et al*., Structures of the HIN domain:DNA complexes reveal ligand binding and activation mechanisms of the AIM2 inflammasome and IFI16 receptor, Immunity. 36 (4), 561–571 (2012).
- Morrone, SR. et al. Cooperative assembly of IFI16 filaments on dsDNA provides insights into host defense strategy. Proc Natl Acad Sci U S A. 111(1):E62-71 (2014).

Reviewer #2 (Remarks to the Author):

The authors have sufficiently addressed my concerns.

Reviewer #3 (Remarks to the Author):

The revised manuscript by Cislaghi et al. shows improved clarity and structure. The conclusions are more nuanced and fit the underlying data. Inclusion of an additional human cell line and the IFI16 complementation into the KO cells is strongly valued. As a general comment, looking at infectious virus instead of genome copies is preferred. Particularly when talking about restriction of viruses. Nonetheless, the changes in genome copies are influenced by IFI16 which is supported by this data. Overall, the authors addressed the raised comments and questions from my side.
